# Slc1a2-mediated Glutamate Transport Promotes Dendritic Cell Immunity through Sema3A-regulated Cytoskeletal Remodeling and Migration

**DOI:** 10.7150/ijbs.131305

**Published:** 2026-05-29

**Authors:** Shanlin Chen, Bei Chen, Gongwei Li, Haoqiang Liu, Qiang Chen, Jiahao Chen, Yuekang Xu, Adila Aipire, Jinyao Li

**Affiliations:** Xinjiang Key Laboratory of Biological Resources and Genetic Engineering, College of Life Science and Technology, Xinjiang University, Urumqi 830017, China.

**Keywords:** dendritic cells, glutamate, Slc1a2, Semaphorin 3A, GTP hydrolases, cytoskeletal remodeling

## Abstract

Dendritic cells (DCs) can activate T cells to trigger sustained antitumor immune responses, a process in which the continuous migration of antigen-loaded DCs from the tumor microenvironment to tumor-draining lymph nodes is critical. DCs have evolved a complex and dynamic regulatory network to mediate their migration to specific locations, with multiple transporters including amino acid transporters reported to participate in this process. Slc1a2 is highly expressed in the nervous system, where it mediates the clearance of extracellular glutamate, primarily in astrocytes; however, its role in the immune system remains unclear. In this study, we showed that activated DCs upregulated Slc1a2 to boost glutamate uptake, which in turn promoted DC functionality and antitumor vaccine potency. Furthermore, glutamate signaling induced Sema3A, which elevated small GTPase signaling pathways (e.g., RhoA/Rac1/Cdc42) and drove dynamic cytoskeletal remodeling, thereby providing the necessary molecular machinery for DC migration during antitumor immunity *in vivo*. We first demonstrated that Slc1a2-mediated glutamate metabolism functioned as a metabolic checkpoint for DC migration and antigen-specific immunity *in vivo*, with the Sema3A/small GTPase axis serving as the core mechanism linking glutamate signaling to cytoskeletal reorganization.

## Introduction

Immune cells are activated when they recognize pathogens or danger signals. This process is accompanied by profound metabolic reprogramming to meet both bioenergetic and biosynthetic demands required for their functional execution 1-3. As a key bridge between innate and adaptive immunity, dendritic cells (DCs) play an irreplaceable and central role in the uptake and processing of antigens to initiate antigen specific T-cell responses 4. Likewise, in the tumor microenvironment (TME), activated DCs capture and present tumor-associated antigens and provide co-stimulatory signals and cytokines to elicit T cell immunity against tumor cells 5. During these process, the metabolic demands in DCs increase dramatically, as evidenced not only by significantly higher consumption of glucose (to support aerobic glycolysis) 1, but also by active and sophisticated regulation of the uptake and utilization of key nutrients such as amino acids 6.

Recent studies have shown that, in addition to serving as substrates for protein synthesis, amino acids are profoundly involved in regulating key immune functions such as DC maturation, cytokine secretion, antigen presentation and migration through various mechanisms including participating in mTOR signaling pathway activation, metabolic reprogramming, redox balance maintenance, and acting as signaling molecules, etc. For example, dysregulated arginine metabolism in DCs, has been correlated with abnormal differentiation of peripheral helper T cells 7. The Th2 cytokines, such as IL-4 and IL-10, represent efficient inducers of a key enzyme expression in the arginine metabolism 8. Glutamate and glutamine can serve not only as a carbon source for the TCA cycle 9 but also as a substrate for the antioxidant-reduced glutathione, which is crucial for maintaining redox homeostasis 10. During tularemia, glutamate utilization promotes rapid mitochondrial ROS production in DCs, driving systemic inflammation^11^. Amino Acid Transporters (AATs), which are localized on the cell membrane, play a crucial role as metabolic gatekeepers in this process. Activated DCs significantly enhance the extracellular amino acid transport flux to the intracellular compartment by specifically up-regulating the expression of various amino acid transporters, thus providing the necessary material basis for the immune activation and effector functions 6, 12. For example, Guo, C. et al. identified an intercellular metabolic crosstalk between tumor cells and cDC1s involving the glutamine transporter SLC38A2, where tumor cells and cDC1s compete for glutamine uptake via SLC38A2 to regulate antitumor immunity, revealing an important role for AATs in antitumor immunity 13. Thus, elucidating how actively regulated amino acids control DC activation and function is key to uncovering immunometabolic principles and developing amino acid-targeted immunotherapies for vaccines/autoimmune disorders.

Activated DCs actively regulate amino acid uptake, obtaining the necessary material for them to perform key immune functions. In the DC-mediated adaptive immune response, their migratory ability plays a central role: it is a prerequisite and a fundamental step for DCs to carry processed antigens from peripheral tissues to secondary lymphoid organs to meet with the T cells for initiation of a specific antitumor immune response 14-16. DC migration is a highly dynamic and precisely regulated process that involves the synergistic action of multiple complex intracellular mechanisms, including post-translational modification of proteins, epigenetic reprogramming, metabolic remodeling, and, critically, cytoskeletal rearrangement 14, 16. Cytoskeletal rearrangements are the central physical basis driving DC morphological changes and directed migration, and the mechanisms behind them are sophisticated and complex 17-19. Upon sensing microenvironmental cues (e.g., chemokines), DCs necessitate dynamic remodeling of actin and microtubule networks—including polymerization, depolymerization, and crosslinking-to form pseudopods, regulate adhesion, and generate directed motility forces 20-22. These energy-intensive cytoskeletal dynamics, driven by ATP hydrolysis, are pivotal for effective DC migration. Critically, the ability of DCs to initiate motility in response to microenvironmental signals relies not only on signal transduction cascades but also on metabolic substrate supply. We thereby hypothesize that amino acid-driven intracellular metabolic reprogramming in activated DCs, coupled with efficient production of energy currencies, collectively establishes an indispensable bioenergetic and biosynthetic foundation for dynamic cytoskeletal remodeling that ultimately underpins DC migration.

In this study, we demonstrated that activated DCs upregulate the amino acid transporter Slc1a2 (Solute carrier family 1 member 2, also known as EAAT2/GLT-1) to enhance glutamate uptake. Investigation into the impact of glutamate on DC functionality revealed that intracellular glutamate supports DC maturation, migration, and antigen specific immunity *in vivo* as readout by antitumor immune responses, with the most pronounced effect observed in migration—a prerequisite for DCs immunocompetence. Mechanistically, glutamate reshapes the cytoskeleton movement through the downstream effector Sema3A, which activates small GTPase signaling pathways (e.g., RhoA/Rac1), thereby powering cytoskeletal remodeling and directional migration. These findings provide novel mechanistic insights into amino acid-mediated regulation of DCs immunity, and have significant implication in DC-mediated immunotherapy.

## Results

### Maturation stimuli up-regulate Slc1a2 for intracellular transportation of Glu in DCs

**Figure 1:**
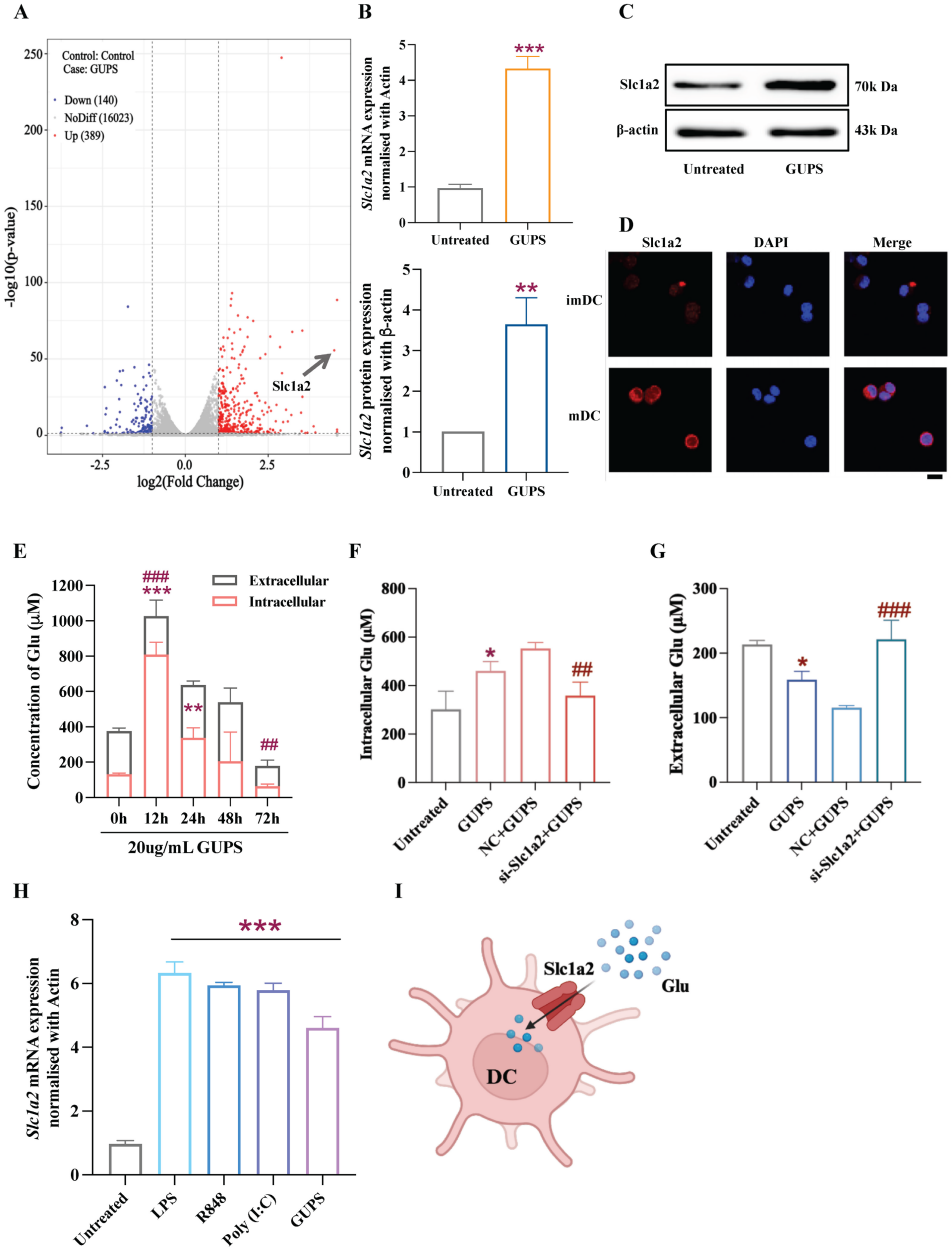
** Activation-stimulated expression of Slc1a2 in mDCs mediates glutamate transport. A** Volcano plot of the differentially expressed mRNAs in BMDCs stimulated with 20 μg/mL *Glycyrrhiza uralensis* polysaccharides (GUPS) for 12 h or left unstimulated (Control). **B** qRT-PCR analysis of gene expression of Slc1a2 in GUPS-activated BMDCs, n=4/group. **C** Western blot analysis of protein expression of Slc1a2 in GUPS-activated BMDCs, n=3/group. **D** immunofluorescence staining revealed the membrane localization of Slc1a2 (red). DNA (blue) was stained with DAPI. Scale bars, 10 μm. **E** Intracellular and extracellular Glu levels after treatment with GUPS at different time points, n=3/group, *: intracellular Glu level, all groups compared to the untreated group, #: extracellular Glu level, all groups compared to the untreated group. **F-G** Intracellular/extracellular Glu levels in BMDCs after siRNA transfection and GUPS-treatment, n=3/group, *: GUPS group compared to untreated group, #: si-Slc1a2+GUPS group compared to NC+GUPS group. **H** qRT-PCR analysis of Slc1a2 gene expression in splenic DCs following different stimuli, n=4/group. **I** Schematic illustrations of DCs depend on Slc1a2 to transport Glu. Data are presented as mean ± SD. Statistical analysis was performed using two-tailed Student's t-test (**B**,** F, G**), One-Way ANOVA (**E**,** H**). ^*^P < 0.05, ^**, ##^P < 0.01, ^***, ###^P < 0.001.

We previously demonstrated that *Glycyrrhiza uralensis* polysaccharides (GUPS) promoted DC maturation and cytokine secretion through the TLR4 signaling pathway, and identified the GUPS as an adjuvant that significantly enhanced the antitumor effect of the HPV-DC vaccine 23. To determine the molecular mechanism of the GUPS-mediated DC maturation, we performed RNA-Seq after treating with or without GUPS and found that the *Slc1a2* gene was one of the three genes significantly upregulated in the GUPS-stimulated matured DCs (mDCs) (p-value=2.43677E-56; fold change=22.52788152;) (Fig. 1A), which was also confirmed by mRNA and protein data (Fig. 1B-C). Since Slc1a2 was reported to be responsible for transporting the excitatory neurotransmitter glutamate (Glu) from the synaptic gap to the intracellular compartment 24-26, we first investigated whether the highly expressed Slc1a2 in the mature DCs (mDCs), has any functional implication in transporting Glu. Confocal assay demonstrated a membrane localization of Slc1a2 in mDCs, providing physical evidence for its possible role as gatekeeper or molecular switch for extracellular Glu (Fig. 1D). Interestingly, when DCs were cultured in the presence of 20 μg/mL GUPS (based on dose response results previously published 23) for 0~72 h, we observed that intracellular Glu levels increased while extracellular Glu levels decreased as DC matured, with peak at 12 h (Fig. 1E). To establish the causal effect of Slc1a2 with the GUPS-induced intracellular Glu transportation, small interfering RNA (siRNA) was used to knock down the expression of Slc1a2, with the most effective siRNA chosen (Fig. S1A) to evaluates the changes between intra- and extracellular Glu contents. The results demonstrated that the deletion of Slc1a2 significantly reduced intracellular Glu levels in the si-Slc1a2+GUPS group compared to nonspecific control siRNA (NC)+GUPS, whereas the opposite was true for extracellular Glu levels, indicating the compromised capacity of DCs to uptake Glu due to the lack of Slc1a2 (Fig. 1F-G). Consistently, overexpression of Slc1a2, on the contrary, was found to promote the Glu uptake by DCs (Fig. S1B), confirming our hypothesis that DCs matured by GUPS upregulate Slc1a2 to transport Glu into the intracellular compartment.

Next, we were curious whether the maturation-associated expression of Slc1a2 and its intracellular transportation of Glu were specific to GUPS stimulation, or generic to all DC maturation/activation stimuli. Therefore, we stimulated splenic cells *in vitro* with LPS and other TLR agonists. Subsequently, we sorted CD11c-positive cells from splenocytes using magnetic beads and assessed Slc1a2 expression levels. The results showed that, compared with the untreated splenic DCs cultured *in vitro* for 24 h, DCs stimulated with LPS, R848 and Poly(I:C) all demonstrated elevated Slc1a2 expression, as was observed with GUPS (Fig. 1H). These results indicate that the maturation-mediated Slc1a2 upregulation is of generic nature, rather than specific to GUPS, and the upregulated Slc1a2 expression mediated Glu transport into intracellular compartments (Fig. 1I).

### Slc1a2 participates in DC function and migration

**Figure 2:**
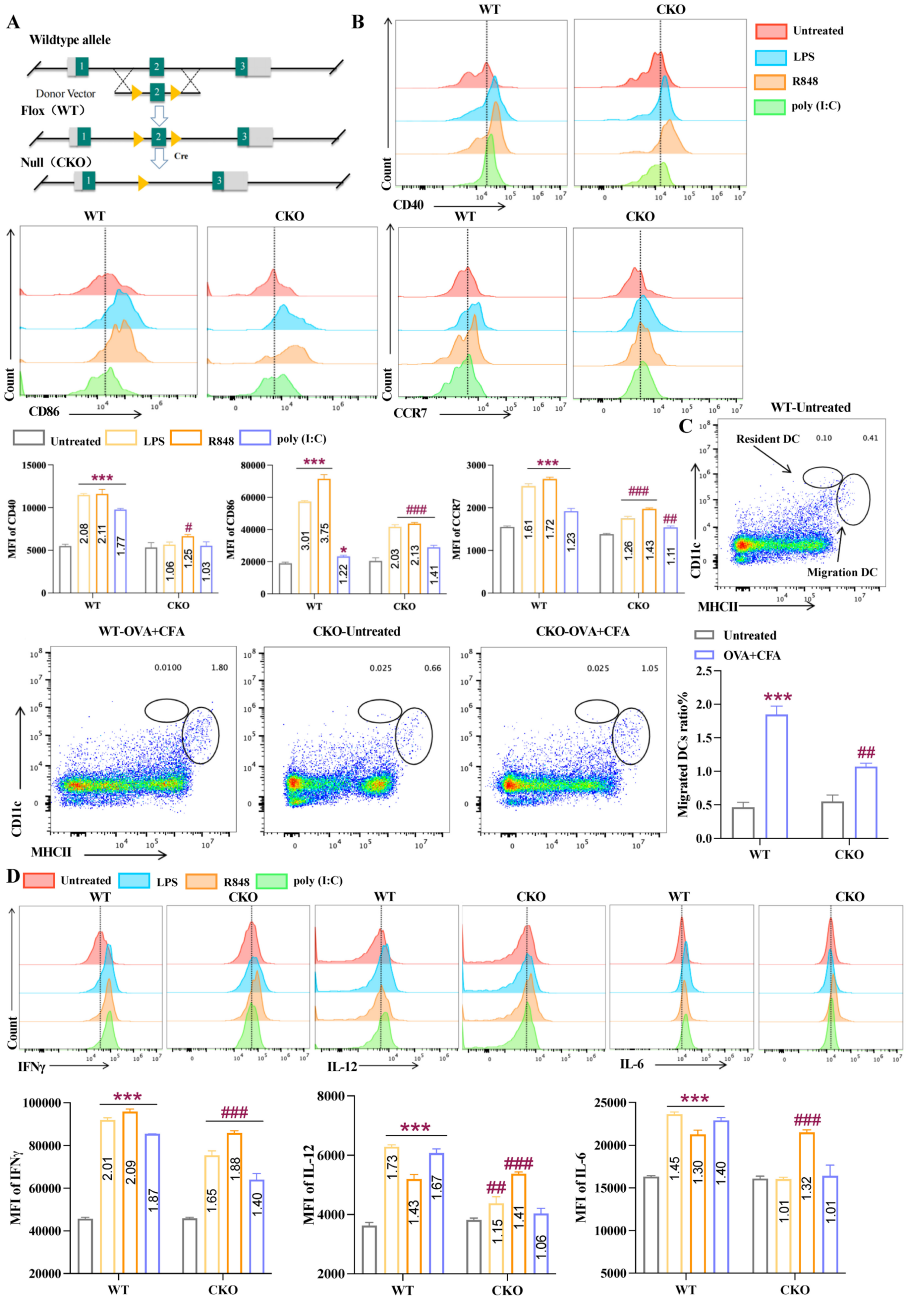
** DCs depend on Slc1a2 for maturation initiation, migration, and cytokine secretion *in vivo*. A** Schematic diagram of the construction of Slc1a2^fl/fl^Itgax-cre^+^ conditional knock-out (CKO) mice using CRISPR-Cas9 technology. **B** TLR agonist-triggered maturation and migration of splenic DCs, analyzed by WT/CKO popliteal lymph nodes, n=3/group. */#: WT/CKO mice, all groups compared to the untreated group. **C** The proportion of resident DCs and migratory DCs in popliteal lymph nodes of WT/CKO mice following OVA immunization, n=3/group. */#: WT/CKO mice, all groups compared to the untreated group. **D** Mean fluorescence intensity (MFI) of IFN-γ, IL-12, and IL-6 in popliteal lymph node DCs following TLR agonist treatment in WT/CKO mice, n=3/group. */#: WT/CKO mice, all groups compared to the untreated group. Data are presented as mean ± SD. Statistical analysis was performed using two-tailed Student's t-test (**C**), One-Way ANOVA (**B**,** D**). ^*, #^P < 0.05, ^##^P < 0.01, ^***, ###^P < 0.001.

To determine the indispensable role of Slc1a2 in DCs, we generated Slc1a2^fl/fl^Itgax-cre^+^ conditional knock-out (CKO) mice with specific deletion of Slc1a2 in CD11c^+^ DCs through CRISPR-Cas9 (Fig. 2A). Since previous studies have revealed that Slc1a2 is highly expressed in the nervous system, we confirmed that the deficiency of Slc1a2 expression in CD11c^+^ cells (whether in splenic DCs or BMDCs) from CKO mice (Fig. S1C-D) without causing pathological damage to their brain tissue (Fig. S1E). In addition, CKO mice organ indicators were normal (Fig. S1F). The frequency of major immune cell subsets in CKO mice remained intact, compared to the Slc1a2^fl/fl^Itgax-cre^-^ mice control (hereinafter referred to as WT) (Fig. S1G-I). Based on the successful construction of CKO mice, we next determined whether Slc1a2 deficiency in DCs affects TLR agonist-triggered innate immune activation, including maturation, migration, and cytokine secretion processes. *In vivo*, following TLR agonist (LPS, R848, Poly(I:C)) injection via the plantar route, analysis of CD11c and MHCII double-positive cells in draining lymph nodes (dLNs) revealed that in WT mice, TLR agonist significantly upregulated the maturation- and migration-associated surface molecules (CD40, CD86, CCR7). Interestingly, CKO mice by comparison, exhibited reduced responsiveness to TLR agonist stimulation, with lower expression of CD40, CD86, and CCR7 (Fig. 2B). After capturing antigens, activated mDCs must be able to rapidly and effectively initiate migration so that they can “meet” with T cells, which is essential for rapidly triggering adaptive immunity 27. To monitor endogenous DC migration, we administered a combined treatment of OVA (100 μg) and CFA via plantar injection in WT/CKO mice. 12 h later, dLNs were collected and cells were released by treatment with collagenase. Migratory (MHC II^high^ CD11c^int^) and resident (MHC II^int^ CD11c^high^) DCs was assessed by flow cytometry. As shown in Figure 2C, compared to the untreated group, OVA-immunized WT mice exhibited a reduced proportion of resident DCs and an increased proportion of migratory DCs, whereas in CKO mice, the upregulation of migratory DCs was significantly less pronounced than in WT mice. Furthermore, we evaluated cytokine secretion in DCs migrated to dLNs following subcutaneous injection of TLR agonists, and the results showed that TLR agonist treatment significantly enhanced IFN-γ, IL-12, and IL-6 secretion in DCs from WT mice. Compared to untreated controls, the fold increase generally exceeded 1.5-fold. However, in CKO mice, this effect was markedly attenuated, with fold increases consistently below 1.5-fold (Fig. 2D). The results of the *in vivo* experiments above indicate that Slc1a2 plays a crucial role in DC maturation, migration, and cytokine secretion.

### Slc1a2 regulates intracellular glutamate levels for the cytoskeleton-dependent migration of DCs

**Figure 3:**
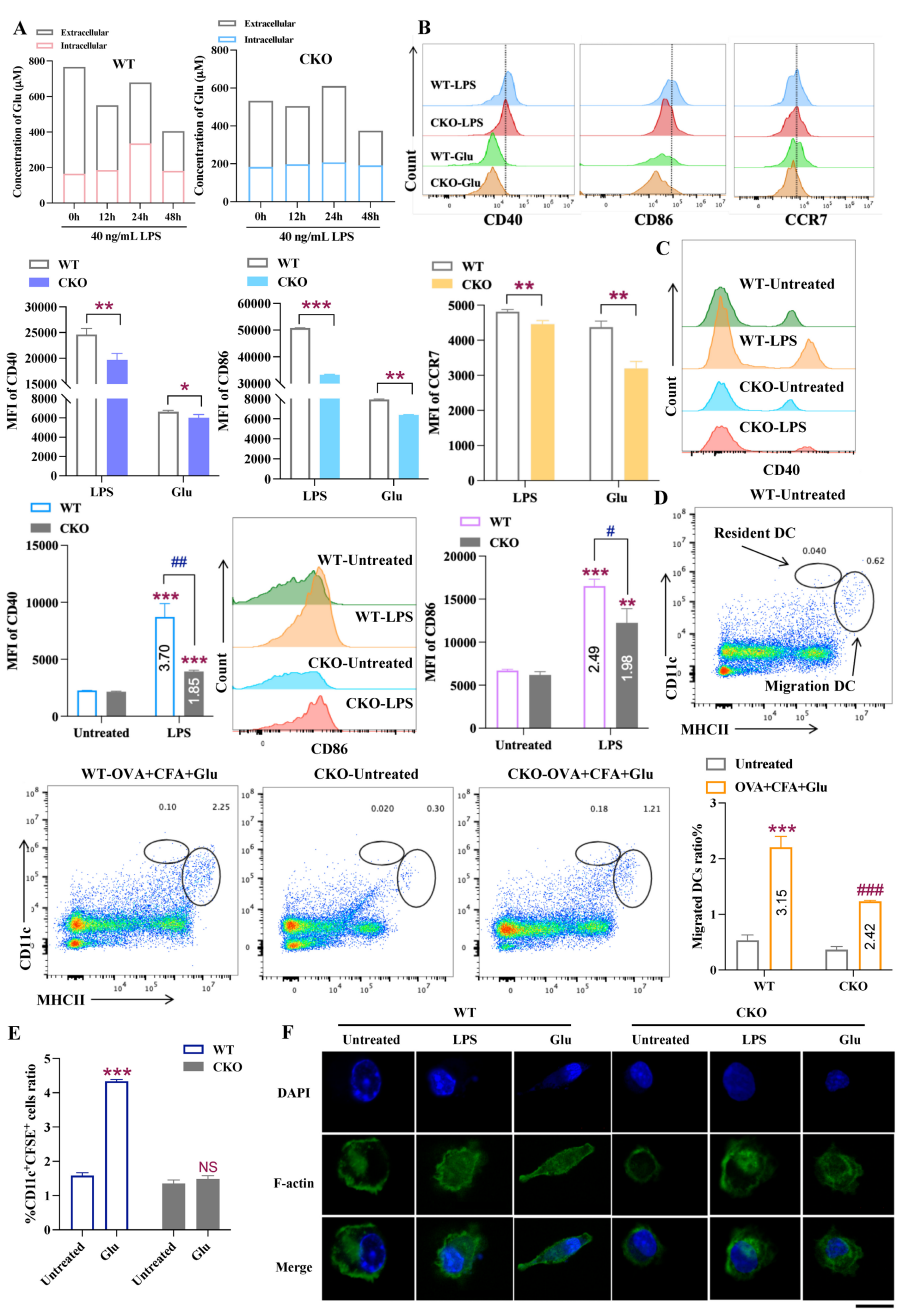
** Slc1a2 mediates Glu-induced DC activation and migration. A** Intracellular and extracellular Glu levels of WT and CKO-derived BMDCs stimulated by LPS at different time points. **B** LPS/Glu-triggered maturation and migration of DCs, analyzed by WT/CKO popliteal lymph nodes, n=3/group. **C** Following intravenous injection of Glu, LPS-triggered maturation of DCs, analyzed by WT/CKO popliteal lymph nodes, n=3/group. *: WT/CKO mice, LPS group compared to the untreated group. #: LPS group, WT and CKO were compared. **D** The proportion of resident DCs and migratory DCs in popliteal lymph nodes of WT/CKO mice following OVA+Glu immunization, n=3/group. */#: WT/CKO mice, all groups compared to the untreated group. **E** Glu-triggered migration of WT/CKO-derived BMDCs, analyzed by popliteal lymph nodes, n=3/group, *: WT-derived DCs, Glu group compared to the untreated group, NS, not significant.** F** immunofluorescence staining revealed the Glu-triggered cytoskeletal remodel in WT/CKO-derived BMDCs, DNA (blue) was stained with DAPI, F-actin (green). Scale bars, 10 μm. Data are presented as mean ± SD. Statistical analysis was performed using two-tailed Student's t-test (**B**,** D**,** E**), Two-Way ANOVA (**C**). ^*, #^P < 0.05, ^**, ##^P < 0.01, ^***, ###^P < 0.001.

Given that Slc1a2 serves as the Glu gatekeeper in DCs and plays a crucial role in DC maturation, migration, and cytokine secretion processes, we next aim to elucidate whether intracellular Glu levels is the mediator of the above roles of Slc1a2 in DCs. We first assessed the Glu transport capacity of BMDCs derived from WT and CKO mice. As shown in Figure 3A, during LPS (40 ng/mL) treatment, intact Slc1a2 in the WT-derived BMDCs exhibited normal capacity to transport Glu intracellularly, while the intracellular Glu levels in CKO-derived BMDCs showed no significant change. Therefore, we hypothesize that the increased intracellular Glu transport by the up-regulated Slc1a2 on mDCs led to their maturation and migration.

To validate this hypothesis, *in vivo*, WT and CKO mice were treated with Glu (150 μg) and LPS (30 μg). Following LPS or Glu injection via the plantar route, analysis of CD11c and MHCII double-positive cells revealed that in WT mice, LPS and Glu significantly upregulated the maturation- and migration-associated surface molecules (CD40, CD86, CCR7). Interestingly, CKO mice by comparison, exhibited reduced responsiveness to both LPS and Glu stimulation, with lower expression of CD40, CD86, and CCR7 (Fig. 3B). In addition to direct Glu sensitization, we administered intravenous Glu (150 μg) to WT and CKO mice to elevate circulating glutamate levels. 6 h post-treatment, LPS was injected via the plantar route. Our finding revealed that reduced intracellular Glu transport flux due to Slc1a2 deficiency in CKO mice resulted in significantly decreased expression of maturation-associated surface molecules CD40 and CD86 upon LPS stimulation compared to WT mice (Fig. 3C). Consistent with previous methods evaluating endogenous DC migration 28, we supplemented OVA+CFA with Glu (150 μg). Results demonstrated that Glu combined with OVA significantly promoted DC migration to dLNs in WT mice, with a substantial increase in the proportion of migratory DCs (MHC II^high^ CD11c^int^) (upregulated by 3.15-fold). Conversely, the increase in migratory DCs in CKO mice was markedly lower than that in WT mice (upregulated by 2.42-fold) (Fig. 3D). Furthermore, measuring the chemokine receptor CCR7, and the number of cells migrating from the plantar to the popliteal lymph nodes in mice demonstrated that the Glu-induced dose-dependent overexpression of CCR7 and increased number of migrating cells were all lost in the CKO-derived BMDCs (Fig. 3E, S3C). Since DC migration is accompanied by cytoskeletal reorganization 29, we also investigated the cytoskeletal changes in Glu-treated BMDCs at the microscopic level by using phalloidin to label F-actin (green) and DAPI to label DNA. As shown in Figure 3F, Glu treatment changed and remodeled the cytoskeleton in WT-derived DCs but not in CKO-derived DCs.

To validate the direct role of Glu in DCs, we treated wild-type BMDCs with Glu* in vitro*, and found that 2 mM treatment induced increased Slc1a2 expression and intracellular Glu levels (Fig. S2A-C). Concurrently, Glu treatment promoted the expression of maturation-associated surface molecules (CD40, CD86, MHCII) and cytokine secretion in the BMDCs (IL-12, TGF-β, IL-6, IL-10, TNF-α) (Fig. S2D-E). Consistently, direct plantar administration of Glu to C57BL/6 mice *in vivo* also resulted in high expression of maturation-associated surface molecules in CD11c^+^ MHCII^+^ cells within dLNs, exhibiting a dose-dependent pattern (Fig. S2F). Furthermore, this maturation process of DCs was accompanied by their decreased phagocytic ability in terms of uptake of FITC-dextran (Fig. S2G). To investigate whether the Glu-induced BMDC maturation could affect their migration ability *in vitro* and* in vivo*, one portion of Glu-matured BMDCs were analyzed *in vitro* using a Transwell chamber, whereas the other portion were labeled with CFSE, injected into the plantar to analyse the number of BMDCs migrating to the popliteal lymph nodes. Interestingly, we observed that Glu had a remarkable effect on promoting DC migration both *in vitro* and *in vivo*, even more pronounced than that of LPS, as evidenced by the highest number of cells migrating to the bottom chamber and popliteal lymph nodes (Fig. S2H-I). Since CCR7 chemokine receptor is strongly associated with DC migration toward dLNs, we further examined CCR7 expression after treating DCs with different concentrations of Glu. The results showed that Glu could upregulate CCR7 expression in a dose-dependent manner (Fig. S2J), suggesting a direct effect of Glu on CCR7 expression.

To further confirm the causal effect of Slc1a2 in the Glu-associated DC activation and migration, we cultured WT/CKO-derived BMDCs *in vitro* and found that WT-derived BMDCs exhibited heightened sensitivity to Glu treatment, upregulating maturation-associated surface molecules (CD40, CD86, MHCII) (Fig. S3A). Concurrently, Glu reduced the phagocytic capacity of WT-derived BMDCs toward FITC-dextran, while having no effect on CKO-derived BMDCs (Fig. S3B). Further, WT-derived BMDCs were transfected or virally transduced to overexpress or knockdown Slc1a2, and the expression of inflammation-related cytokines was determined using ELISA. As shown in Figure S4A, Slc1a2 knockdown significantly inhibited the increase in IL-6, IL-12, and TNF-α, which was previously induced by Glu (Fig. S2E). Conversely, overexpression of Slc1a2 promoted the expression of these cytokines (Fig. S4B). Furthermore, we also examined CCR7 expression as a readout of DC migration. Consistent with ELISA results, knockdown of Slc1a2 inhibited Glu-induced high expression of CCR7, while overexpression of Slc1a2 promoted the CCR7 expression (Fig. S4C). We next asked whether the upregulation of Slc1a2 upon TLR engagement renders DC activation sensitive to the availability of extracellular Glu. To test this, we stimulated WT/CKO-derived BMDCs with TLR agonists (LPS, Poly(I:C), CpG, R848) in Glu-deprived medium supplemented with defined concentrations of Glu (0, 1, and 2 mM). In WT-derived BMDCs, under Glu-free conditions (0 mM), TLR agonists induced only a modest increase in surface maturation markers (CD40 and CD86). Supplementation with 1 mM Glu significantly enhanced the TLR agonists-induced upregulation of CD40 and CD86, and 2 mM Glu further amplified this response, yielding a clear dose-dependent effect. In striking contrast, CKO-derived BMDCs showed no significant changes in the expression of maturation markers in response to stimulation by most TLRs under Glu (0, 1, and 2 mM): the expression levels of both CD40 and CD86 remained relatively low (Fig. S5). These results demonstrate that Slc1a2-mediated Glu transport enhances TLR-induced DC maturation in a dose-dependent manner.

We also prepared a liposome-encapsulated Glu (Lipo-Glu) delivery system, which simultaneously treated WT/CKO-derived BMDCs. Result indicated that Glu delivered via liposomes remains functional in Slc1a2-deficient CKO mice, as evidenced by increased expression of maturation-associated surface molecules, with no significant differences observed compared to WT-derived BMDCs (Fig. S6), this finding further confirmed the role of Slc1a2 as the Glu “gatekeeper.” Collectively, these results suggested that Slc1a2-mediated intracellular Glu transport regulates DC maturation and migration.

Notably, as a sodium-dependent, high-affinity amino acid transporter, Slc1a2 also mediates the transport of aspartate (Asp) 30-32. To determine whether the observed effects were specific to Glu rather than a general amino acid-mediated response, we treated WT/CKO-derived BMDCs with Asp at increasing concentrations. In WT-derived BMDCs, at concentration equivalent to Glu (2 mM), Asp failed to induce DC maturation, CCR7 expression, or cytoskeletal remodeling. Notably, even at a higher concentration (10 mM), Asp only modestly increased CCR7, without recapitulating the full spectrum of glutamate-induced effects, and remained markedly less effective than 2 mM Glu (Fig. S7). In contrast, neither Glu nor 10 mM Asp induced any such effects in CKO-derived BMDCs. These results indicate that Glu exerts a more potent and functionally comprehensive effect on DC activation, supporting a ligand-specific signaling mechanism rather than a generic amino acid-dependent response.

### Slc1a2-mediated intracellular glutamate transport in DCs improves T cell immunity in the host

**Figure 4:**
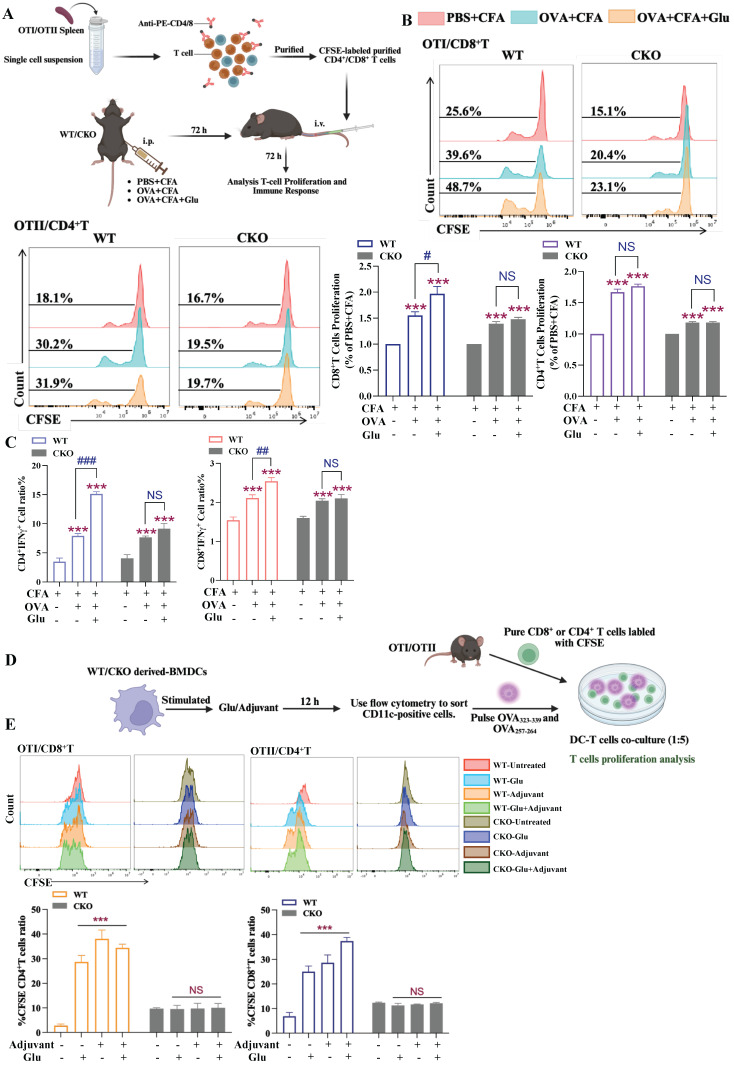
** Slc1a2 mediates Glu-induced improved T cells immune responses. A** Schematic diagram for detecting DC-T cell interaction *in vivo*. **B** CD4^+^ or CD8^+^ T cell CFSE dye dilution in WT/CKO mouse spleens, n=3/group, *: all groups compared to the PBS+CFA group, #: OVA+CFA group compared to the OVA+CFA+Glu group, NS, not significant. **C** Proportion of CTL and Th1 cells in WT/CKO mouse spleens, n=3/group, *: all groups compared to the PBS+CFA group, #: OVA+CFA group compared to the OVA+CFA+Glu group, NS, not significant. **D** Schematic diagram for detecting DC-T cell interaction *in vitro*. **E** CD4^+^/CD8^+^ T cell CFSE dye dilution in DC-T cell interaction system *in vitro*, n=3/group, *: WT-derived DCs, all groups compared to the untreated group, NS, not significant. Data are presented as mean ± SD. Statistical analysis was performed using One-Way ANOVA (**E**), Two-Way ANOVA (**B**, **C**). ^#^P < 0.05, ^##^P < 0.01, ^***, ###^P < 0.001.

With the evidence of Slc1a2-regulated DC maturation and migration, we next want to examine the effect of this Glu gatekeeper on the DC-mediated T cell immunity *in vivo*. To this end, we isolated and purified splenic T cells from OTI/OTII mice, labeled them with CFSE, and then intravenously injected them into WT/CKO mice pre-immunized with OVA (CFA/Glu as adjuvant). After 72 h, mice spleens were isolated to assess the antigen-specific activation of the adoptively transferred T cells as shown in Figure 4A. We found that the levels of proliferation of the transferred T cells *in vivo* in the peritoneal-OVA+CFA immunized group was 1.5 times higher than that in the PBS+CFA group, indicating antigen specific immune responses by DCs were successfully induced *in vivo*. T cell proliferation induced by OVA+CFA combined with Glu was twice that of the PBS+CFA group, indicating that Glu can further promote OVA-induced DC immunity. In contrast, in CKO mice, although OVA immunization promoted the antigen specific T cell proliferation compared to the PBS+CFA group, the enhanced effect of Glu on the OVA-induced immunity was lost, as there was no significant difference in immune responses elicited between OVA+CFA and OVA+CFA+Glu treatments (Fig. 4B). In line with the T cell proliferation profiles, treatment with OVA+CFA+Glu in WT mice induced the highest levels of Th1 cell (CD4^+^ IFNγ^+^) and CTL cell (CD8^+^ IFNγ^+^) activation, whereas no significant difference was observed between OVA+CFA+Glu and OVA+CFA in the Slc1a2 CKO mice (Fig. 4C).

In line with the findings *in vivo*, FACS-sorted CD11c^+^ cells were pulsed with OVA antigen, and co-cultured with CSFE labelled CD4^+^/CD8^+^ T cells for 72 h *in vitro* (Fig. 4D). The results showed that WT-derived DCs stimulated to maturity significantly promoted the proliferation of CD4^+^ and CD8^+^ T cells. Notably, CD8^+^ T cell proliferation was higher in the Adjuvant+Glu group than in the Adjuvant group, indicating Glu enhances the adjuvant effects. In contrast, CKO-derived DCs showed no significant differences in T cell proliferation (neither CD4^+^ nor CD8^+^ T cells) compared to the untreated group, indicating that DC-mediated T cell proliferation depends on Slc1a2 (Fig. 4E).

**Figure 5:**
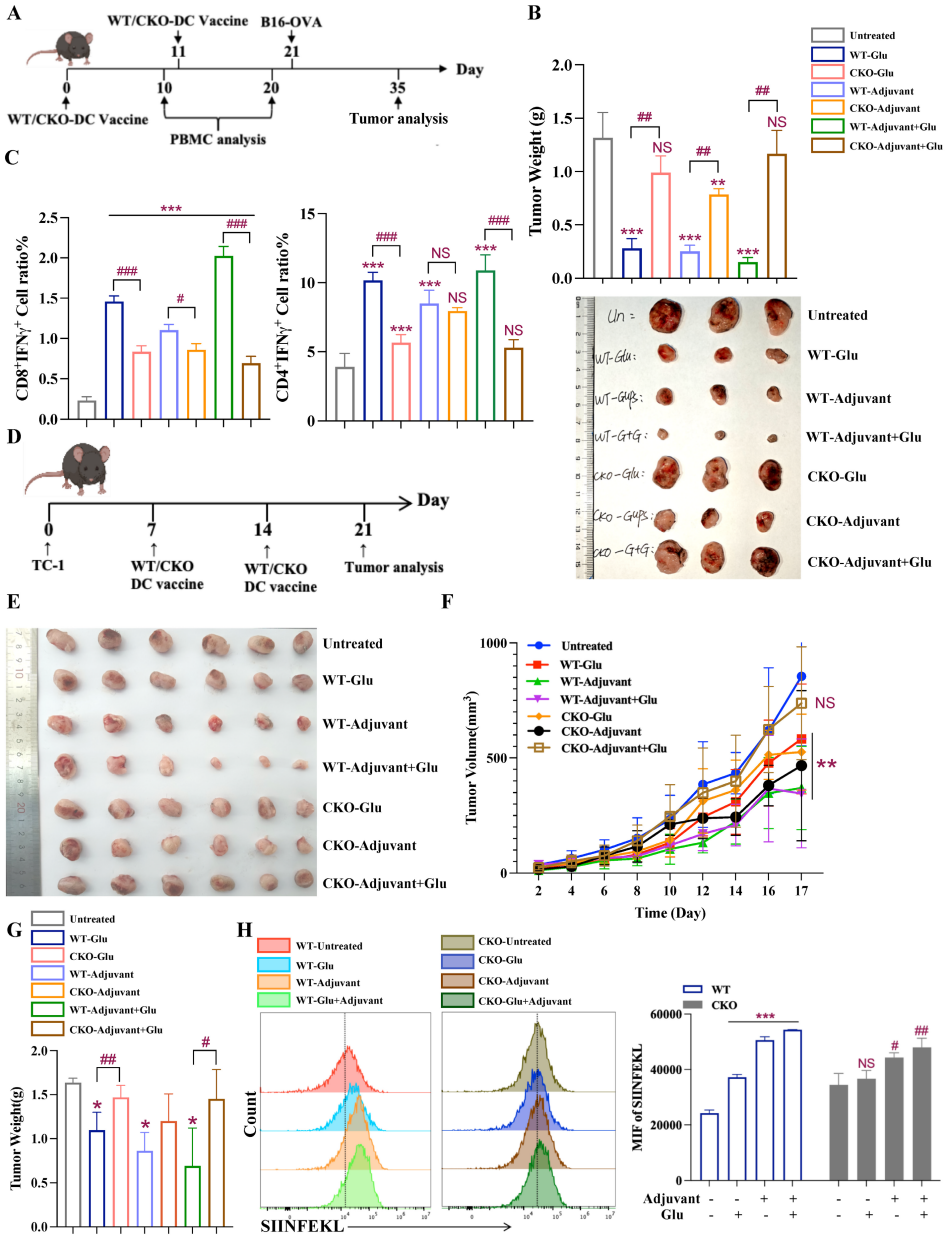
** Slc1a2 mediates Glu-induced better antitumor immune responses. A** Timeframe for WT/CKO-derived DC preventive vaccines effect in the B16-OVA tumor model, n=3/group. **B** Tumor volume images and tumor weight data for each treatment group are given, *: all groups compared to the untreated group, #: under the same treatment, WT and CKO-derived BMDC vaccines were compared, NS, not significant.** C** Proportion of CTL and Th1 cells in PBMC after the second treatment with WT/CKO-derived BMDC vaccines, n=3/group, *: all groups compared to the untreated group, #: under the same treatment, WT and CKO-derived BMDC vaccines were compared, NS, not significant. **D** Timeframe for WT/CKO-derived BMDC therapeutic vaccines effect in the TC-1 tumor model, n=6/group. **E, G** Tumor volume images and tumor weight data for each treatment group are given, *: all groups compared to the untreated group, #: under the same treatment, WT and CKO-derived BMDC vaccines were compared.** F** Tumor growth in the TC-1 model was estimated in the indicated groups, *: all groups compared to the untreated group, NS, not significant. **H** Following stimulation with OVA, WT/CKO-derived SIINFEKL^+^ DCs and MFI of SIINFEKL were evaluated, n=3/group, */#: WT/CKO-derived BMDCs, all groups compared to the untreated group, NS, not significant. Data are presented as mean ± SD. Statistical analysis was performed using two-tailed Student's t-test and One-Way ANOVA (**B**, **C**, **G**), One-Way ANOVA (**H**), Two-way Repeated Measures ANOVA (**F**). ^*, #^P < 0.05, ^**, ##^P < 0.01, ^***, ###^P < 0.001.

We next investigated in a diseased setting whether CKO-derived DCs pulsed with antigens would show weakened antigen-specific antitumor response *in vivo* in comparison with WT-derived DCs. BMDCs were activated in the same way as *in vitro* assay, followed by treatment with OVA_323-339_ and OVA_257-264_ peptides for 2 h, before they were injected into C57BL/6 mice on days 0 and 11. B16-OVA cells were subcutaneously inoculated on day 21, and tumor growth trends were assessed on day 35, as illustrated in Figure 5A. Figure 5B shows tumor volume and weight, revealing that the WT-derived DC vaccines exhibited significantly stronger antitumor efficacy compared to the CKO-derived vaccines. To evaluate antigen-specific immune responses, after one shot of DC vaccination, PBMCs were isolated and analyzed for activated CD4^+^ (CD44^+^ CD4^+^) and CD8^+^ (CD44^+^ CD8^+^) cells, as well as Th1 (CD4^+^ IFNγ^+^) and CTL (CD8^+^ IFNγ^+^) cells (Fig. S9A-D). After the second DC vaccine treatment, the proportions of Th1 cells (CD4^+^ IFNγ^+^) and CTL cells (CD8^+^ IFNγ^+^) in Figure 5C and Figure S9E demonstrated that WT DCs induced a higher level of immune response than CKO DCs under stimulus treatment. These results showed that stimulant-activated WT DC vaccines restricted tumor growth and significantly increased the proportion of CD4^+^IFNγ^+^ and CD8^+^IFNγ^+^ subsets, whereas these abilities were lost in CKO DC vaccines.

To rule out the antigen-dependent factors, we also evaluated the antitumor efficacy of DC specific Slc1a2 in TC-1(cell lines stably expressing HPV antigens) tumor-bearing mice. First, TC-1 cells were injected subcutaneously into C57BL/6 mice on day 0. Subsequently, DCs from WT/CKO mice were treated with Glu, Adjuvant, and Adjuvant+Glu for 12 h, followed by loading with HPV peptides for 2 h. DC vaccines were administered on days 7 and 14, and tumor growth trends were analyzed on day 21 (Fig. 5D). The results showed that, WT DC vaccines exhibited better antitumor effects than those of CKO, as evidenced by reduced tumor volumes and weights (Fig. 5E-G).

Since antigen cross-presentation is the key element in DC mediated antitumor immunity, we treated WT/CKO-derived DCs with OVA antigen for 2 h after Glu, and used monoclonal antibody specifically against the H-2Kb-bound SIINFEKL peptide to measure the MHCI-presented OVA peptide as a readout of cross-presentation capacity. The result showed that Glu treatment of the WT DCs triggered stronger cross-presentation compared to CKO DCs (Fig. 5H).

Collectively, these results above indicated that Slc1a2-dependent Glu transport in DCs initiated an improved T cell immune response *in vivo*.

### RNA-Seq reveals the Semaphorin family responds to intracellular glutamate signaling

**Figure 6:**
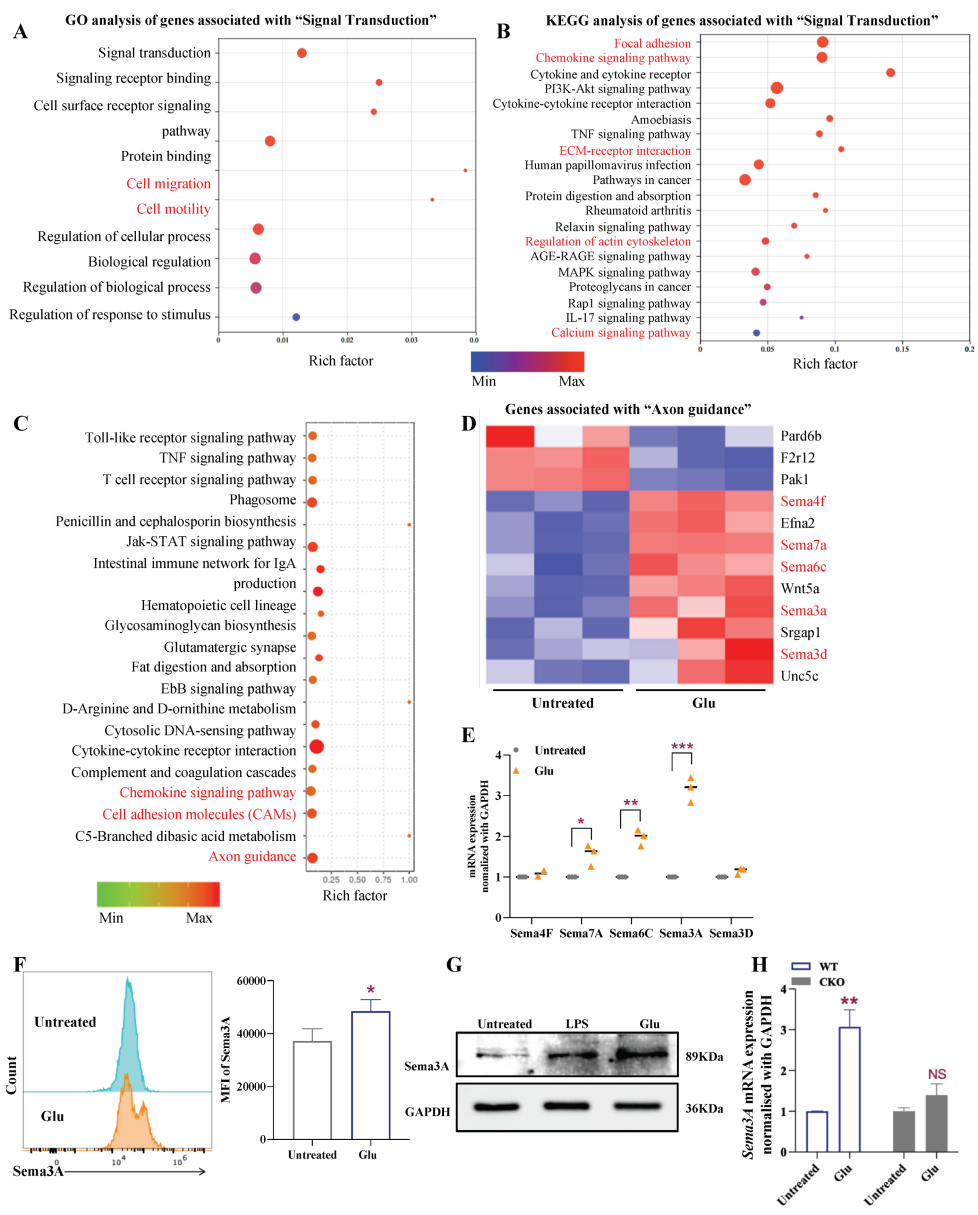
** Sema3A is the major targets of intracellular Glu in DCs. A-B** GO (**A**) and KEGG (**B**) analyzed the differentially expressed mRNAs with and without Glu treatment. **C** KEGG analysis revealed the axon guidance pathways were enriched after BMDCs treated with GUPS. **D** cluster analysis of genes in axon guidance pathways in (**C**).** E** qRT-PCR analysis of Sema family members mRNA expression after Glu treatment, n=4/group. **F, G** flow cytometry and western blot analysis the expression of Sema3A in Glu-activated BMDCs, n=3/group. **H** qRT-PCR analysis of Sema3A mRNA expression after Glu treatment in WT/CKO-derived BMDCs, n=4/group, NS, not significant. Data are presented as mean ± SD. Statistical analysis was performed using two-tailed Student's t-test. ^*^P < 0.05, ^**^P < 0.01, ^***^P < 0.001.

To identify the intracellular target of Glu-mediated DC immunity *in vivo*, we performed RNA-Seq of BMDCs after treatment with or without Glu. The results indicated that the intracellular Glu-triggered pathways were related to cell migration and cell motility, such as focal adhesion, chemokine signaling pathway, in which extracellular matrix (ECM)-receptor interaction, and regulation of actin cytoskeleton were particularly enriched (Fig. 6A-B). Among them, the Semaphorin (Sema) family in the axon guidance pathway caught our attention (Fig. 6C-D), as they were identified as axon growth guidance factors and play an important role in cell chemotaxis, migration 33, and regulation of immune cell function 34-36. qRT-PCR analysis of the 5 upregulated Sema family members confirmed that Sema3A was upregulated the most significantly after Glu stimulation (Fig. 6E). Furthermore, we validated the Glu-induced upregulation of Sema3A through flow cytometry and Western blot analysis (Fig. 6F, G). Interestingly, the upregulation of Sema3A by Glu also depended on Slc1a2 (Fig. 6H). As consistent with the previous discussion, the regulatory effect of Sema3A is also Glu-specific, rather than a generic amino acid sensing, a phenomenon that we did not observe in the Asp-treated DC (Fig. S8). Since Sema3A can exhibit chemotactic activity, enabling cells to migrate in a specific direction, and is associated with tumor growth and angiogenesis 35, we speculated that Glu-triggered DC migration may be related to Sema3A.

### Sema3A participated in glutamate signal transduction to initiate DC cytoskeletal remodeling and migration

**Figure 7:**
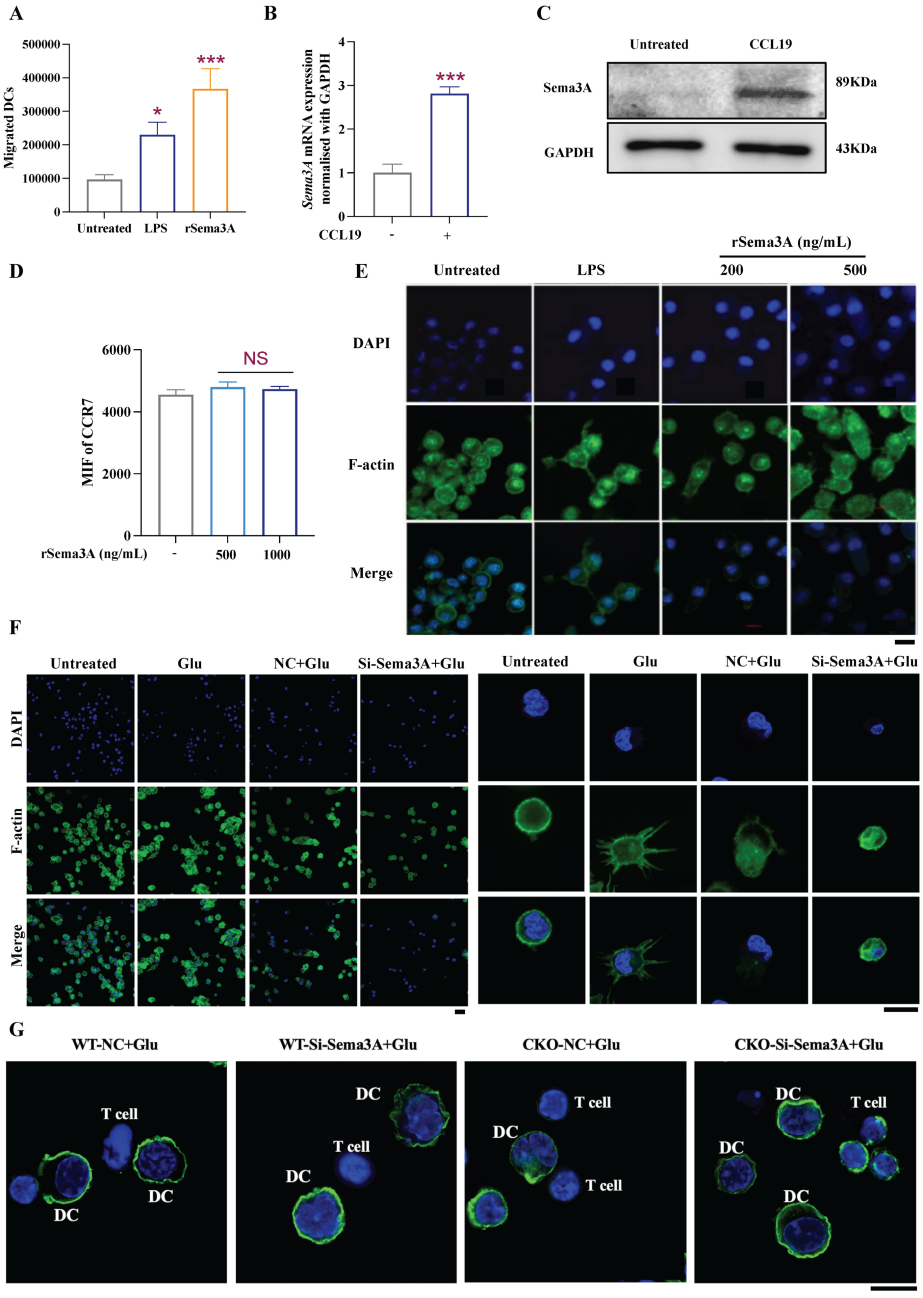
** Sema3A affects DC cytoskeletal changes and migration. A** Transwell chambers analysis of rSema3A-triggered DC migration, n=3/group. **B, C** qRT-PCR and western blot analysis the expression of Sema3A under CCL19 treatment. n=3/group. **D** Flow cytometry analysis of CCR7 expression under rSema3A treatment. n=3/group, NS, not significant.** E, F** immunofluorescence staining revealed the rSema3A-triggered cytoskeletal remodel (**E**), and Glu, si-Sema3A-triggered cytoskeletal remodel (**F**). The right side of **F** is a local enlarged image. DNA (blue) was stained with DAPI, F-actin (green). Scale bars, 10 μm. **G** Confocal imaging revealed DC-T cell interactions and synapses in WT/CKO-derived DCs following Glu and si-Sema3A treatment, DNA (blue) was stained with DAPI, F-actin (green). Scale bars, 10 μm. Data are presented as mean ± SD. Statistical analysis was performed using two-tailed Student's t-test (**B**), One-Way ANOVA (**A**, **D**). ^*^P < 0.05, ^***^P < 0.001.

To verify whether Sema3A can promote DC migration, we prepared recombinant Sema3A protein (rSema3A), and found that exogenous rSema3A treatment promoted the migration of DCs in Transwell chambers (Fig. 7A). In addition, we found that the chemokine CCL19 regulates the expression of Sema3A, but rSema3A cannot upregulate CCR7, suggesting that Sema3A may be downstream of the CCL19/CCR7 signal (Fig. 7B-D). Since the migration of DCs is accompanied by cytoskeletal remodeling, consistently, treatment with rSema3A was found to alter the cytoskeletal morphology of DCs in a dose-dependent manner (Fig. 7E). Next, we observed that cytoskeletal remodeling was reduced in the si-Sema3A+Glu group compared to the negative control (NC) +Glu group (Fig. 7F). It was revealed that Glu-induced cytoskeletal remodeling is achieved by targeting Sema3A. Furthermore, we utilized WT/CKO-derived DCs treated with Glu and transfected with si-Sema3A, which were then co-cultured with sorted CD4^+^ T cells. Confocal imaging revealed DC-T cell interactions and synapses. As shown in Figure 7G, Glu-treated DCs exhibited cytoskeletal remodeling, with WT-derived DCs demonstrating more pronounced synapse formation compared to CKO-derived DCs. Compared to the si-Sema3A+Glu group, the NC+Glu group exhibited more pronounced DC-T clustering. Specifically, due to the absence of Sema3A, Glu no longer induced morphological changes in DCs nor recruited DCs to aggregate toward T cells. This suggests that Glu-initiated DC cytoskeletal remodeling, migration and interactions with T cells was dependent on Sema3A.

### Glutamate-mediated Sema3A initiates immune responses by targeting GTPase pathways

**Figure 8:**
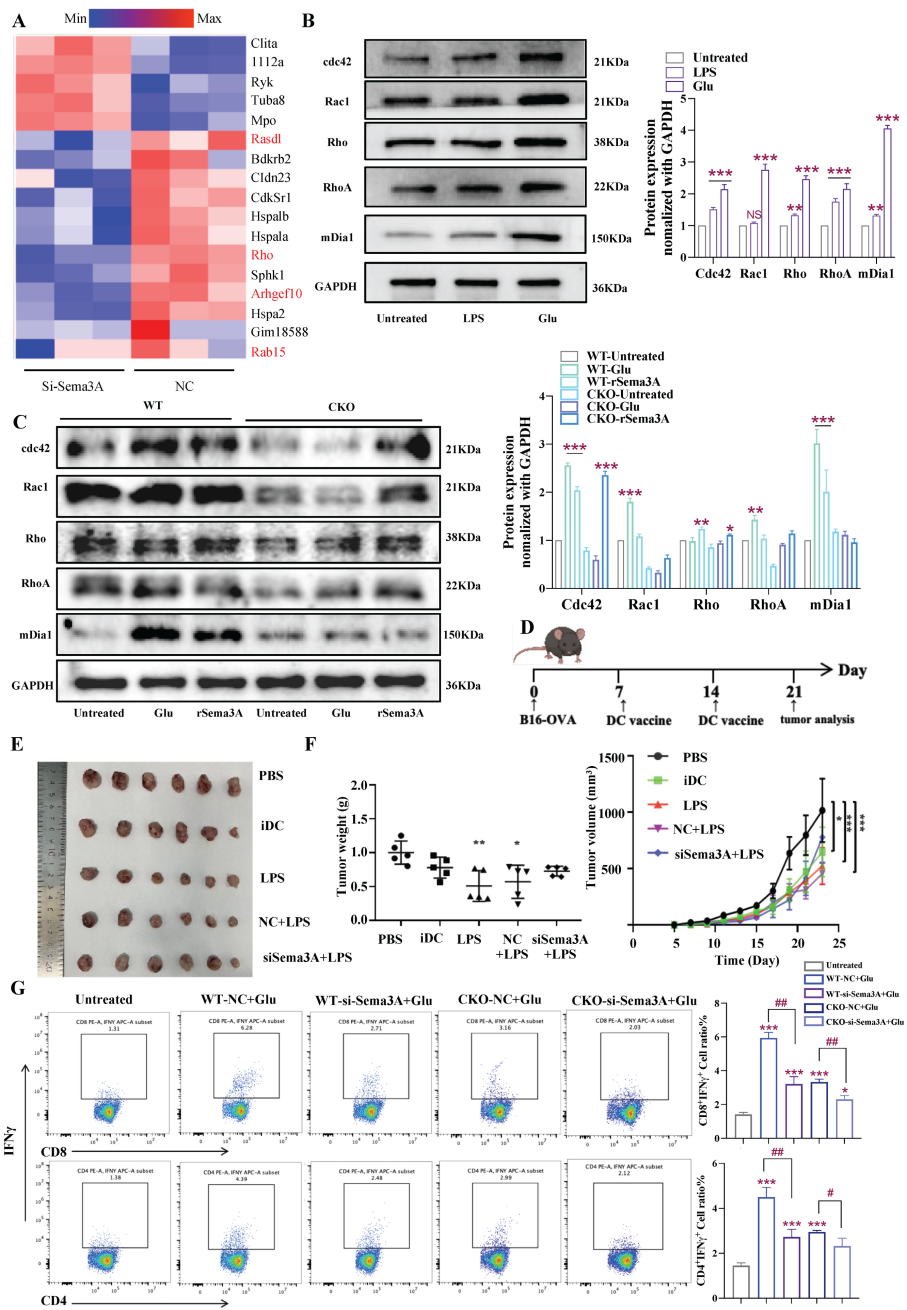
** mDCs initiate antitumor immune responses through the Sema3A/GTPase pathway. A** Heatmaps of the differentially expressed mRNAs in BMDCs transfected with si-Sema3A or NC. **B** Expression of GTPase pathway-related proteins in BMDCs treated with LPS/Glu, n=3/group, *: all groups compared to the untreated group, NS, not significant. **C** Expression of GTPase pathway-related proteins in WT/CKO derived BMDCs treated with Glu/rSema3A, n=3/group, *: all groups compared to the untreated group. **D** Timeframe for DC vaccine effect in the B16-OVA tumor model, n=6/group. **E, F** Tumor growth in the B16-OVA model was estimated in the indicated groups, n=6/group, *: all groups compared to the PBS group. **G** WT/CKO BMDCs stimulated with Glu and transfected si-Sema3A were co-cultured with CD4^+^/CD8^+^ T cells *in vitro* and assessed with the proportion of CTL and Th1 cells, n=3/group, *: all groups compared to the untreated group, #: si-Sema3A+Glu group compared to the NC+Glu group. Data are presented as mean ± SD. Statistical analysis was performed using One-Way ANOVA (**B**,** C**), two-tailed Student's t-test and One-Way ANOVA (**G**), Two-way Repeated Measures ANOVA (**F**). ^*, #^ P < 0.05, ^**, ##^P < 0.01, ^***^P < 0.001.

The Rho small GTPase (Rac, Cdc42, and RhoA) signaling pathway is an important regulator in controlling the acto-myosin cytoskeleton essential for DC migration 37, 38, and Sema3A is a secreted protein that regulate the multiple small GTPases 39, 40. Following transient knockdown of Sema3A, we conducted RNA-Seq analysis, and found that genes associated with the Rho and small GTPase signaling pathways were suppressed in the si-Sema3A group compared to the NC group (Fig. 8A, marked by the red). We examined the expression of Rho, Cdc42, Rac1, RhoA, and mDia1 and found that this Rho GTPase pathway was also upregulated upon stimulation with LPS and Glu (Fig. 8B). To further dissect the regulatory hierarchy among Slc1a2, Glu, and Sema3A, we treated WT/CKO-derived DCs with Glu or rSema3A (Fig. 8C). In WT DCs, both Glu and rSema3A upregulated the expression of these GTPase proteins. In contrast, in CKO DCs, Glu failed to induce GTPases upregulation, whereas rSema3A remained fully effective. These data indicated that Glu upregulated the GTPase pathway through Sema3A, and that loss of Slc1a2 abolished this Glu-dependent effect, which could be rescued by supplementation with rSema3A.

Next, we explored whether the Sema3A-mediated cytoskeletal remodeling and Rho/GTPase pathway elevation in DCs were key regulatory hubs that initiated the antitumor immune responses. By constructing the B16-OVA melanoma model in C57BL/6 mice, we investigated the antitumor effects of antigen specific vaccines prepared from immature DCs (iDCs), mature DCs (LPS-activated DCs), and DCs transfected with si-Sema3A or si-NC (Fig. 8D). The results showed that treatment with DC vaccines significantly reduced tumor weight and growth compared with the untreated group (PBS). Furthermore, among the various vaccine groups, mature DC vaccines demonstrated better therapeutic effects (iDC vs LPS). When comparing the si-Sema3A and NC groups, the absence of Sema3A significantly weakened the antitumor effect of the DC vaccines (Fig. 8E-F). This suggests that Sema3A is indispensable for mature DCs to initiate antitumor immune responses *in vivo*.

Finally, to identify the mechanisms of the antitumor effect of Sema3A-mediated DC vaccines, and determine whether Sema3A is a downstream target of Glu-activated DCs, we used purified DC-T co-culture system *in vitro*. As shown in Figure 8H, consistent with previous results, Glu promotes an increased proportion of DC-induced CTL (CD8^+^ IFNγ^+^) and Th1 cell subsets (CD4^+^ IFNγ^+^) when Slc1a2 remained intact, but this function was impaired in the Sema3A-deficient DCs. In CKO-derived DCs, we found that neither Glu treatment nor Sema3A deficiency could induce an increase in the proportion of CTL and Th1 cell subsets (Fig. 8G). Together, these data highlighted the value of Sema3A in response to Slc1a2-controlled Glu transport in regulating DC migration and subsequent adaptive immune activation.

## Discussion

In present study, we identified Slc1a2-mediated glutamate (Glu) uptake as a critical regulator of DC-driven adaptive immunity. The core mechanism underpinning Glu's role involves orchestrating cytoskeletal remodeling in DCs—an essential physical infrastructure enabling both migration and immunological functions of DCs, including antigen presentation and T cell activation 15, 41, 42. We define Sema3A as a Glu-inducible rheostat that licenses DC motility through GTPase-driven cytoskeletal dynamics (RhoA/Rac1/Cdc42). These findings unveil an amino acid-directed regulatory axis in rewiring DC immunometabolism, fundamentally advancing mechanistic paradigms in myeloid cell biology.

It has been previously reported that extracellular Glu in tumor immune microenvironment (TIME) is able to act as a signaling molecule on Glu receptors on the surface of T-cells and tumor cells, activating downstream signaling cascades and regulating tumor immune escape 43-45. However, its role in DCs remain elusive. Here, we demonstrated for the first time that Glu in DC functioned beyond conventional metabolic roles—not merely as an energy substrate or protein synthesis precursor—but as a potent signaling molecule to direct DC cytoskeleton remodeling and migration that directly governed their induction of adaptive immunity *in vivo*. Previous studies have also reported that the availability of amino acids directly affects the organizational structure of the cytoskeleton. In cancer cells, glutamine deprivation has been shown to decrease Rac1-GTP and Cdc42-GTP levels while altering cofilin and profilin balance, thereby impairing directional migration and invasiveness^46^. Similarly, in macrophages genetic deletion of glutaminase (Gls1) significantly suppresses Cdc42 and Rac1 activity, leading to defective actin polymerization^47^. Despite these advances, the specific molecular pathway from amino acid transport to the cytoskeletal machinery required for DC migration had not been established. Our results showed that the requirement of Glu was substantially increased in mDCs, and Glu transport to the intracellular compartment supported DC maturation, migration, and antitumor immune responses via Slc1a2, which is consistent with the notion that immune cells compete with tumor cells for glutamine in the tumor microenvironment 13. Specifically, Glu-activated DCs showed significant activation of maturation-associated phenotypes and the vaccines prepared from them exerted better antitumor efficacy *in vivo*. Interestingly, Glu transport mediated by Slc1a2 triggered significant DC migration. It is well known that DCs can upregulate the expression of chemokine receptor CCR7 in response to chemokine signaling and activate downstream related events to initiate migration. Along this line, we found that CCR7 was highly expressed following the treatment of Glu, and their migration efficiency increased with cytoskeleton remodeled. In contrast, Slc1a2-deficient DCs showed reduced migration efficiency and difficulty in exerting antitumor effects due to Glu deficiency, suggesting that Slc1a2 transported, Glu-mediated cytoskeleton remodeling and migration enhancement are the main pathways for DCs to promote antitumor immunity.

Cytoskeletal remodeling critically underpins DC functions—including migration, immune synapse formation, endocytosis/exocytosis, and environmental adaptation through morphological plasticity. Our study directly couples Glu metabolism to this fundamental cellular process, bridging for the first time the knowledge gap between metabolic signaling and cytoskeletal dynamics—with Sema3A emerging as the pivotal molecular hub. For example, our RNA-Seq results revealed the importance of Sema3A in migration, cell motility, and adhesion events, and its ability to respond to chemokine signaling, with high expression following CCL19 stimulation. Furthermore, under the treatment of recombinant Sema3A, both DC migration and cytoskeleton remodeling were enhanced, demonstrating its close association with DC migration. Interestingly, Glu-activated DCs exhibited changes in cytoskeleton and the elevation in protein levels of signaling molecules associated with cytoskeleton remodeling like small GTPases: RhoA, Rac1, Cdc42, Rho, mDia1, and all of the cytoskeleton remodeling-associated events were deprived in the absence of Sema3A, indicating that the effect of Glu on DC migration is achieved by targeting Sema3A. Our findings suggested that the upregulation of total Rac1, Cdc42, Rho, RhoA and mDia1 proteins in response to Sema3A or Glu treatment may enhance the potential for GTPase activation. Although functional activation remains to be directly measured, the observed changes in cytoskeletal dynamics and cell migration support the involvement of these GTPases in the signaling processes. Future experiments, including GTP pull-down assays, will provide more direct evidence for the activation of these GTPases and their role in regulating cellular responses. Since migration is the cornerstone for DCs to initiate adaptive immune responses* in vivo*, we further demonstrated that in tumor-bearing mouse models Sema3A is critical for Glu-activated DC vaccines to exert antitumor effects.

Our present study on nutrient metabolism-mediated regulation of DC immunity have profoundly advanced our understanding of the significance of DC immunometabolism. Within this paradigm, we specifically elucidated the role of Glu and its dedicated transporter Slc1a2 in orchestrating DC immune functions—particularly via migration and cytoskeletal dynamics. Although Asp shares the same transporter (Slc1a2), it required substantially higher concentrations to elicit only partial effects, highlighting a clear difference in signaling potency and suggesting that Glu acts as a preferential and functionally dominant ligand in this context. This work addresses a critical gap by establishing the mechanistic link between amino acid metabolism and cellular motility machinery. Previous studies revealed that Sema3A inhibited T cell activation and regulated macrophages in the immune system 48-50. Our study, however, revealed a novel, positive pro-migratory and immune supportive roles of Glu/Sema3A in DCs, enriching the understanding of their immunomodulatory functions. Furthermore, in the TME, as the accelerated glutamine metabolism in tumor cells leads to high levels of Glu, we speculated that it might have a regulatory effect on the tumor-infiltrating DCs, and that the upregulation of Slc1a2 by DCs might be a strategy for them to take advantage of the high Glu environment in the TIME for adaptive antitumor responses. Along this line, promoting Glu/Slc1a2/Sema3A pathway in DCs could have great therapeutic values.

Collectively, the present study uncovered the Slc1a2-mediated Glu uptake as critical metabolic support for activated DC functions, with pronounced effects on motility. We further delineated a novel Sema3A-small GTPase regulatory axis that directly controlled cytoskeletal remodeling. Targeting the Slc1a2/Sema3A/GTPase axis may represent a promising therapeutic strategy to enhance DC vaccine efficacy or improve DC infiltration and functionality within TMEs.

## Materials and Methods

### Mice

All mice were on the C57BL/6 background. Female mice were used for the TC-1 tumor model, while male mice were used for the primary BMDCs and B16-OVA tumor models. Wild-type C57BL/6 mice were purchased from Xinjiang Medical University (Urumqi, China). OT-I and OT-II transgenic mice were sourced from Jackson Laboratory (United States) and housed under specific pathogen-free (SPF) conditions at Xinjiang University. Slc1a2^fl/fl^ (Mice carrying the loxP-targeted Slc1a2 gene), and H11-Itgax-iCre mice (Strain NO. T004819) were purchased from GemPharmatech (Nanjing, China). Slc1a2^fl/fl^ mice were crossed with Itgax-cre^+^ mice to generate CD11c^+^ cell-specific Slc1a2-knockout mice (Slc1a2^fl/fl^Itgax-cre^+^, abbreviated to CKO). Age- and sex-matched littermate controls (Slc1a2^fl/fl^Itgax-cre^-^, abbreviated to WT) were used for all experiments involving conditional knockout mice. Animals were randomly assigned to experimental groups based on body weight. All animal procedures were approved by and performed according to Animal Experimental Ethics Committee of Xinjiang University's Laboratory Animal Centre (Approval No. XJUAE-2023-012).

### Cell purification and culture

Bone marrow cells were extracted from the tibia and femur of C57BL/6 mice and cultured in RPMI medium containing GM-CSF (10 ng/ml) as previously described 51. On day 6 of culture, BMDCs were collected and prepared into a cell suspension with a concentration of 0.5-2×10⁷ cells per ml for FACS sorting. Next, surface staining was performed using anti-CD11c-FITC (Elabscience, Cat: E-AB-F0991C). The percentage of CD11c^+^ cells was calculated based on live cells. With unstained samples serving as blank controls, cells within the CD11c^+^ gate were collected, and the resulting CD11c^+^ cells were designated as immature DCs (imDCs). imDCs were further stimulated with LPS (40 ng/mL) (Sigma-Aldrich, Cat: L4391) , GUPS (20 μg/mL) (We isolated and purified in our previous study 23), Glu (2mM) (Solarbio, Cat:IG0710), in some cases, Poly(I:C) (5 μg/mL) (MedChemExpress, Cat: HY-107202) or R848 (5 μM) (MedChemExpress, Cat: HY-13740) for 12 h to generate mature DCs (mDCs). After OT-I/OT-II mice were euthanized, their spleens were prepared into a single-cell suspension and stained with anti-CD8-PE (Elabscience, Cat: E-AB-F1104D) and anti-CD4-PE (Elabscience, Cat: E-AB-F1097D), respectively. After positive selection and separation with anti-PE microbeads (Miltenyi Biotec, Cat: 130-048-801), the collected cells were CD4^+^ and CD8^+^ T cells. B16F10-OVA and TC-1 cells were purchased from the Cell Bank of Chinese Academy of Science (Shanghai, China) and used for constructing tumor model.

### Flow cytometry

For cell surface staining, the single-cell suspensions were incubated with the antibody cocktails for 20 min at room temperature away from light. For intracellular cytokine staining, cells were fixed with 4% paraformaldehyde at 4 °C for 30 minutes, washed with cleaning solution, and then stained with a cytokine antibody cocktail at room temperature in the dark for 20 minutes. Data were acquired on a Beckman Coulter flow cytometer and analyzed with FlowJo software.

### DC maturation *in vivo* and *in vitro*

For *in vivo* experiments, PBS, LPS (50 μg), Glu (150 μg), Poly(I:C) (100 μg), R848 (30 μg) were injected into the mouse plantar. 12 h later, the popliteal lymph nodes were isolated and prepared into a single-cell suspension. Subsequently, as previously described, co-stained with anti-CD11c-FITC, anti-CD40-PE, anti-CD86-APC and anti-MHC Class II-PE-Cyanine7 antibodies according to the procedure of the manufacturer, and then examined by flow cytometry.

For *in vitro* experiments, BMDCs cultured for 6 days were seeded at 1 × 10⁶ cells/well in a 24-well culture plate, and Glu (2 mM), LPS (40 ng/mL) was added to the cell culture system. Cells were harvested 12 h later. Co-stained with anti-CD11c-FITC (Elabscience, Cat: E-AB-F0991C), anti-CD40-PE (eBioscience, Cat: 12-0401-83), anti-CD86-APC (eBioscience, Cat: 17-0862) and anti-MHC Class II-PE-Cyanine7 (eBioscience, Cat: 25-5321-82) antibodies according to the procedure of the manufacturer, and then examined by flow cytometry.

### Antigen uptake by DCs

The internalization of FITC-dextran (Invitrogen, Cat: D1823) was detected by flow cytometry to indirectly reflect DC maturation based on phagocytic capacity. mDCs were collected and FITC-dextran (1 mg/mL) was added. After incubation at 37°C for 1 h, the reaction was terminated with cold PBS. After three washes, the cells were resuspended in PBS. FITC-dextran uptake by mDCs was detected by flow cytometry.

### DC migration *in vitro*

The expression level of CCR7 on the surface of mDCs at least partially reflects their migratory capacity. Anti-CCR7-Percp (BD Pharmingen, Cat: 560812) was co-stained with mDCs, and flow cytometry was used to analyze CCR7 expression.

Transwell migration assays were performed using 24-well Transwell plates containing 8-μm-pore polycarbonate filters (Corning, Life Science). 100 ng/ml CCL19 (R&D systems, Cat: 440-m3-025) was added to the lower chambers (total volume is 500 μL). mDCs were added to the upper chamber (1×10^5^ cells in a total volume of 100 μL) and incubated for 6 h at 37°C. The number of migrated DCs into the lower chamber was determined by flow cytometry.

### mDC migration *in vivo*

For endogenous periphery DC migration, WT/CKO mice were intradermally injected with OVA+CFA or OVA+CFA+Glu, 12 h after injection, Gating scheme of migratory (MHCII^high^ CD11c^int^) and resident (MHCII^int^ CD11c^high^) DCs in the draining lymph nodes by flow cytometry. The percentages of migratory DCs and resident DCs in the draining lymph nodes were quantitated by flow cytometry.

For exogenous BMDC migration, 1×10⁷ cells/mL Glu/LPS activated-BMDCs were added to 1 μL of 1 mM CFSE (eBioscience, Cat: 65-0850-84) and stained at 37 °C for 10 minutes. In some experiments, DCs were derived from WT or CKO mice. A total of 2×10^6^ CFSE-labeled DCs was injected subcutaneously into the plantar of C57BL/6 mice. 12 h after DC administration, popliteal lymph nodes were collected and the proportion of CFSE^+^ CD11c^+^ DCs was evaluated by flow cytometry.

### Immunofluorescence staining

mDCs were seeded at 5×10⁵ cells per well in confocal dishes and cultured overnight at 37°C with 5% CO_2_, then, the cells were washed three times with PBS without calcium and magnesium, fixed with 4% paraformaldehyde for 20 minutes, permeabilized with 0.1% Triton X-100 (Solarbio, Cat: IR9073) for 10 minutes, and blocked with 5% BSA (Solarbio, Cat: SW3015) for 1 h. Next, incubate overnight with phalloidin-FITC (Beyotime, Cat: C1033) or Slc1a2 primary antibody (Abcam, Cat: ab205248). The antibody is localized using a biotin-labeled secondary antibody (Cy3-labeled anti-rabbit IgG, Servicebio, Cat: GB27303). DAPI was used to stain nuclei (blue). Confocal images of stained BMDCs were analyzed with an inverted microscope (Carl Zeiss, Göttingen, Germany) using the Zeiss LSM program. All scale bars represent 10 μm, although physical bar length varies with magnification.

### RT-qPCR

Total RNA was extracted from cultured cells with Foregene cell total RNA isolation kit (Foregene, Cat: RE-03113) according to manufacturer's instructions. Reverse transcriptional PCR was performed using the PrimeScript™ RT reagent Kit with gDNA Eraser (Takara, Cat: RR047A) and TB Green® Premix Ex Taq™ II (Takara, Cat: RR820A), according to the manufacturer's suggestions. mRNA expression was normalized to the expression of GAPDH. Results are then relative to those in the Untreated group, set as 1.

The RT-qPCR primer sequences used are as follows:

*GAPDH* Forward: AGGTCGGTGTGAACGGATTTG, *GAPDH* Reverse: GGGGTCGTTGATGGCAACA, *Slc1a2* Forward: TATGCCCAAGCAGGTAGAAGTGC, *Slc1a2* Reverse: TATCAACATGACCACATCAGGGTGG, *Sema4F* Forward: TTATGCTTGCGAGTGTCAGGAG, *Sema4F* Reverse: TCAGGAGGAGAGTGAGAGATGC, *Sema7A* Forward: CAGGGTGGTGAGAGTTCGT, *Sema7A* Reverse: GTCAGGGAGCAGGAAGACA, *Sema6C* Forward: CGCTCTCTTTGGGGTCTTC, *Sema6C* Reverse: CTCCTTGAACTTGCCCTCA, *Sema3A* Forward: ACGGGACTTCGCTATCTTC, *Sema3A* Reverse: CTTTGTCATCTTCAGGGTTGT, *Sema3D* Forward: CAAGGTCTCTGGGGCTAATGC, *Sema3D* Reverse: TCTGGATTATAGGTGTCTGGAATGG.

### Western blot

Cell protein extracts were prepared by utilizing RIPA lysis buffer (Beyotime, Cat: P0013B), supplemented with protease inhibitor cocktail (MedChemExpress, Cat: HY-K0010). Protein concentrations were determined with bicinchoninic acid assay (Thermo Scientific, Cat: 23235).

After the proteins are fully denatured, 15-30 μg of protein from each sample was loaded onto a polyacrylamide gel with a gel concentration of 8-12%. Separate the protein samples on the concentrating gel and separating gel under a constant current of 80 V for 90 minutes. After transferring the protein bands from the gel to a methanol-activated PVDF membrane using a transfer apparatus, the membrane was blocked with 5% milk at 37 °C for 2 h. The primary antibody was incubated overnight at 4 °C. The secondary antibody was incubated at room temperature for 1 h. Protein bands were detected using the Mini Chemiluminescent Imaging and Analysis System (Bio-Rad ChemiDoc MP) chemiluminescent gel imaging system. Primary and secondary antibodies used in this study are listed as follows. Primary antibodies: Anti-EAAT2 (Slc1a2) (Rabbit mAb antibody) (Abcam, Cat: ab205248), Anti-β-actin (Rabbit mAb antibody) (Servicebio, Cat: GB15003-100), Anti-GAPDH (Rabbit mAb antibody) (Servicebio, Cat: GB15004-100), Anti-Semaphorin 3A (Rabbit mAb antibody) (Abcam, Cat: ab199475), Anti-Rac1(Rabbit pAb antibody) (Abcam, Cat: ab97732), Anti-cdc42 (Rabbit pAb antibody) (proteintech, Cat: 10155-1-AP), Anti-Rho (Rabbit mAb antibody) (Abcam, Cat: ab40673), Anti-RhoA (Rabbit mAb antibody) (Abcam, Cat: ab187027), Anti- mDia1 (Rabbit pAb antibody) (proteintech, Cat: 20624-1-AP). Secondary antibody: HRP conjugated Goat Anti-Rabbit IgG (H+L) (Servicebio, Cat: GB23303).

### ELISA

Collect the culture supernatant of mDCs and examined the cytokine content, including mouse IL-12 (Elabscience, Cat: E-EL-M3062), mouse TGF-β (Elabscience, Cat: E-EL-0162), mouse IL-6 (Elabscience, Cat: E-EL-M0044), mouse IL-10 (Elabscience, Cat: E-EL-M0046) and mouse TNF-α (Elabscience, Cat: E-EL-M3063), the detection methods are all performed according to the manufacturer's protocols.

### Transfection of FUW-Slc1a2 Overexpression Plasmid or si-RNA

The recombinant vectors encoding Slc1a2 and its respective fragments were constructed by PCR-based amplification from mDCs cDNA, followed by subcloning into the FUW expression vector. And the siRNA designed and synthesized by Shanghai Biosune Co., Ltd (Shanghai, China). has the following sequence:

*si-Slc1a2* Forward: GCUGGAACUUUGCCUGUUATT, *si-Slc1a2* Reverse: UAACAGGCAAAGUUCCAGCAC, *si-Sema3A* Forward: GGAGCAGCAACAAGUGGAATT, *si-Sema3A* Reverse: UUCCACUUGUUGCUGCUCCTT, *NC* Forward: ACGUGACACGUUCGGAGAATT, *NC* Reverse: UUCUCCGAACGUGUCACGUTT.

The transfection of FUW-Slc1a2 overexpression plasmid and siRNA interference was performed according to the manufacturer's instructions. Briefly, imDCs were seeded in 24-well culture plate at a density of 4×10^5^ cells per well. Then, siRNA/NC or FUW-Slc1a2/FUW were transfected using Lipofectamine 3000 transfection reagent (Thermofisher Scientific, Cat: L3000015). After 24 h of transfection, imDCs were activated with LPS, Glu, etc., and further cultured for 12 h before subsequent detection.

### Glutamate level Assay

Intracellular (cell harvesting and ultrasonic disruption), extracellular (cell culture supernatant), and glutamate levels in mouse serum were measured using a glutamate content assay kit produced by Beijing Boxbio Science & Technology Co., Ltd. (Cat: AKAM002M). The detection principle of this kit is as follows: Glutamate dehydrogenase (GDH) catalyzes the conversion of glutamate and NAD into α-ketoglutarate, NADH, and NH₄⁺. NADH can be reduced by WST-1 through the hydrogen transfer action of PMS, producing methylthionine. The product has a characteristic absorption peak at 450 nm, and the OD value can be used to reflect the glutamate content.

### Co-culture of DCs and OT-I/II cells

For *in vitro* experiments, OT-I and II cells were purified as described above. Next, the cells were prepared into a 1×10^7^ cells/mL suspension, 1 μL of 1 mM CFSE was added, and stained at 37 °C for 10 minutes. After washing three times with cold PBS, 1 × 10⁶ cells were seeded into a 48-well culture plate. mDCs pulsed with OVA_323-339_ and OVA_257-264_ peptides (MedChemExpress, Cat: HY-P0286 and HY-P1489) (10 μg/ml for each peptide) at 37 °C for 2 h, then incubated with CFSE-stained OT-I or II cells (5/1 T cell/DC ratio) for three days. The effects of mDCs on T cell proliferation and differentiation were detected by flow cytometry.

For *in vivo* experiments, OT-I and OT-II cells were purified and CFSE-labeled as described above. Subsequently, WT/CKO mice received intraperitoneal immunizations with PBS+CFA (Complete Freund's Adjuvant (Thermo Scientific, Cat: 77140)), CFA+OVA, or CFA+OVA+Glu. 72 h later, the aforementioned OT-I and OT-II cells were administered intravenously at 1 × 10⁶ cells per mouse. After an additional 72 h, spleens were harvested and analyzed by flow cytometry to assess DC activation and T cell response levels.

### DC vaccines (preventive and therapeutic) preparation

DC vaccines were prepared according to a previous description 52. Briefly, for prophylactic tumor study, on day 6 of culture, BMDCs were treated with adjuvant, Glu, and adjuvant +Glu for 12 h. After washing with cold PBS, cells were pulsed with OVA_323-339_ and OVA_257-264_ peptides at 37 °C for 2 h. Male C57BL/6 mice (5-7 weeks) were divided into seven groups: Untreated (treated with PBS), WT/CKO-Adjuvant (WT or CKO-derived BMDCs stimulated with adjuvant and pulsing with antigen to prepare DC vaccines), WT/CKO-Glu (WT or CKO-derived BMDCs stimulated with Glu and pulsing with antigen to prepare DC vaccines), WT/CKO-adjuvant+Glu (WT or CKO-derived BMDCs stimulated with adjuvant and Glu in combination, pulsing with antigen, and prepared as DC vaccines), DC vaccines (1 × 10⁶ per mouse) were subcutaneously injected into the flank of mice on days 0 and 11. On day 10 or 20, blood was collected for analysis of immune cells in PBMCs. On day 21, mice received a subcutaneous injection of 2 × 10⁵ B16-OVA cells, followed by close monitoring of tumor growth and weight changes. The tumor volume was calculated using the formula (length)×(width)^2^/2, where the long axis diameter was regarded as the length and the short axis diameter was regarded as the width. Mice were euthanized when the largest diameter of tumors reaches 20 mm, and the ipsilateral inguinal lymph nodes were isolated and prepared into a single-cell suspension for flow cytometry analysis of the immune response level.

For therapeutic tumor study, in TC-1 tumor model, BMDCs cultured to day 6 were treated with adjuvant, Glu, and adjuvant+Glu for 12 h. After washing with cold PBS, cells were pulsed with HPV-16 E6 and E7 peptides (Synthesized by Shanghai Kept Bio) (10 μg/ml for each peptide) at 37°C for 2 h, the peptides included E6_43-57_ (QLLRREVYDFAFRDL), E6_53-62_ (AFRDLCIVYR), E7_11-20_ (YMLDLQPETT), E7_44-62_ (QAEPDRAHYNIVTFCCKCD) and E7_81-94_ (DLLMGTLGIVCPIC). Female C57BL/6 mice (5-7 weeks old) were divided into seven groups: Untreated, WT/CKO-adjuvant, WT/CKO-Glu, WT/CKO-adjuvant+Glu, as described above. TC-1 cells (2×10^5^ cells per mouse) were subcutaneously injected into the upper right flank of C57BL/6 mice on day 0. In B16/OVA tumor model, BMDCs cultured to day 6 were transfected with NC or si-Sema3A and stimulated with LPS, then pulsed with OVA antigen at 37 °C for 2 h. Male C57BL/6 mice (5-7 weeks old) were divided into five groups: PBS, iDCs (immature BMDCs pulsing with OVA antigen, and prepared as DC vaccines), LPS (LPS-activated BMDCs pulsing with OVA antigen, and prepared as DC vaccines), NC+LPS (NC-transfected BMDCs activated by LPS, and pulsing with OVA antigen, prepared as DC vaccines), si-Sema3A+LPS (si-Sema3A-transfected BMDCs activated by LPS, and pulsing with OVA antigen, prepared as DC vaccines), B16/OVA cells (2×10^5^ cells per mouse) were subcutaneously injected into the upper right flank of C57BL/6 mice on day 0. On day 7 and 14, DC vaccines were subcutaneously injected into the lower right flank of mice. Tumor growth and body weight of animals were closely monitored. The tumor volume was calculated using the formula (length)×(width)^2^/2, where the long axis diameter was regarded as the length and the short axis diameter regarded as the width. Mice were euthanized when the largest diameter of tumors reaches 20 mm. In some studies, the ipsilateral inguinal lymph nodes were isolated and prepared into a single-cell suspension for flow cytometry analysis of the immune response status.

### Antigen presentation by DCs

On day 6 of culture, stimulate WT or CKO-derived BMDCs with Glu/adjuvant, using imDCs as a control. Then mDCs incubated with OVA protein (MedChemExpress, Cat: HY-W250978) for 12 h. The expressed SIINFEKL on the surface of DCs was detected by anti-MHCI-SIINFEKL (eBioscience, Cat: 12-5743-82) via flow cytometry.

### RNA-seq data analysis

Total RNA was isolated from control immature BMDCs and mature BMDCs stimulated with Glu for 12 h. In addition, there were also Sema3A-silencing and control NC groups for RNA-Seq, and subjected to cDNA library construction and RNA sequencing by Meiji Biotech Co., Ltd., (Shanghai, China). In brief, mRNA sequencing was conducted using NovaSeq X Plus platform and library construction was carried out using the Illumina NovaSeq Reagent Kit. RSEM 53 was used to quantify gene abundances. Essentially, differential expression analysis was performed using the DESeq2 54 or DEGseq 55. In addition, functional- enrichment analysis including GO and KEGG were performed to identify which DEGs were significantly enriched in GO terms and metabolic pathways at Bonferroni-corrected P-value ≤ 0.05 compared with the whole-transcriptome background. GO functional enrichment and KEGG pathway analysis were carried out by Goatools and KOBAS 56, respectively.

### Hematoxylin-Eosin (HE) Staining

The procedures were conducted as described previously 57, 58. WT and CKO mice brain tissues were dissected and fixed in 4% paraformaldehyde. After being embedded in paraffin and cut into slices, the brain tissue specimens were stained with HE for observing the histological changes.

### Statistical analysis

Data are expressed as mean ± SD. Statistical significance was determined by two-tailed Student's t-test for comparisons between two groups, One-way ANOVA for single-factor comparisons involving three or more groups, Two-way ANOVA for experiments with two independent variables, and Two-way repeated measures ANOVA for longitudinal tumor growth data. Data were analyzed using GraphPad Prism 10 (USA). P values < 0.05 were considered significant (^*, #^P < 0.05, ^**, ##^P < 0.01, ^***, ###^P < 0.001).

## Supplementary Material

Supplementary figures.

## Abbreviations

AATs: Amino Acid Transporters
Asp: Aspartate
BMDCs: Bone Marrow-Derived Dendritic Cells
Cdc42: Cell Division Cycle 42
CFA: Complete Freund's Adjuvant
CFSE: Carboxyfluorescein Succinimidyl Ester
CKO: Conditional Knockout (Slc1a2^fl/fl^Itgax-cre^+^)
CTL: Cytotoxic T Lymphocyte
DCs: Dendritic Cells
dLNs: Draining Lymph Nodes
ECM: Extracellular Matrix
ELISA: Enzyme-Linked Immunosorbent Assay
FITC: Fluorescein Isothiocyanate
Glu: Glutamate
GM-CSF: Granulocyte-Macrophage Colony-Stimulating Factor
GTPase: Guanosine Triphosphate Hydrolase
GUPS: *Glycyrrhiza uralensis* Polysaccharides
IFN-γ: Interferon-Gamma
IL: Interleukin
imDCs: Immature Dendritic Cells
Lipo-Glu: Liposome-Encapsulated Glutamate
LPS: Lipopolysaccharide
mDCs: Mature Dendritic Cells
mDia1: Mammalian Diaphanous-related formin 1
MFI: Mean Fluorescence Intensity
NC: Negative Control
OT-I: OVA-Specific CD8^+^ T Cell Receptor Transgenic
OT-II: OVA-Specific CD4^+^ T Cell Receptor Transgenic
OVA: Ovalbumin
PBMCs: Peripheral Blood Mononuclear Cells
qRT-PCR: Quantitative Real-Time Polymerase Chain Reaction
Rac1: Ras-Related C3 Botulinum Toxin Substrate 1
RhoA: Ras Homolog Family Member A
rSema3A: Recombinant Semaphorin 3A
Sema3A: Semaphorin 3A
Slc1a2: Solute Carrier Family 1 Member 2
TGF-β: Transforming Growth Factor-Beta
TLR: Toll-Like Receptor
TNF-α: Tumor Necrosis Factor-Alpha
WT: Wild-Type (Slc1a2^fl/fl^Itgax-cre^-^)
