# Supplementary material for: Slc1a2-mediated Glutamate Transport Promotes Dendritic Cell Immunity through Sema3A-regulated Cytoskeletal Remodeling and Migration
Source: Int J Biol Sci. 2026 May 29;22(11):5799–821. doi: 10.7150/ijbs.131305 (PMC13282749; doi:10.7150/ijbs.131305)
Supplement: Supplementary file 1 — Supplementary figures. [file ijbsv22p5799s1.pdf]

Supplementary Figures

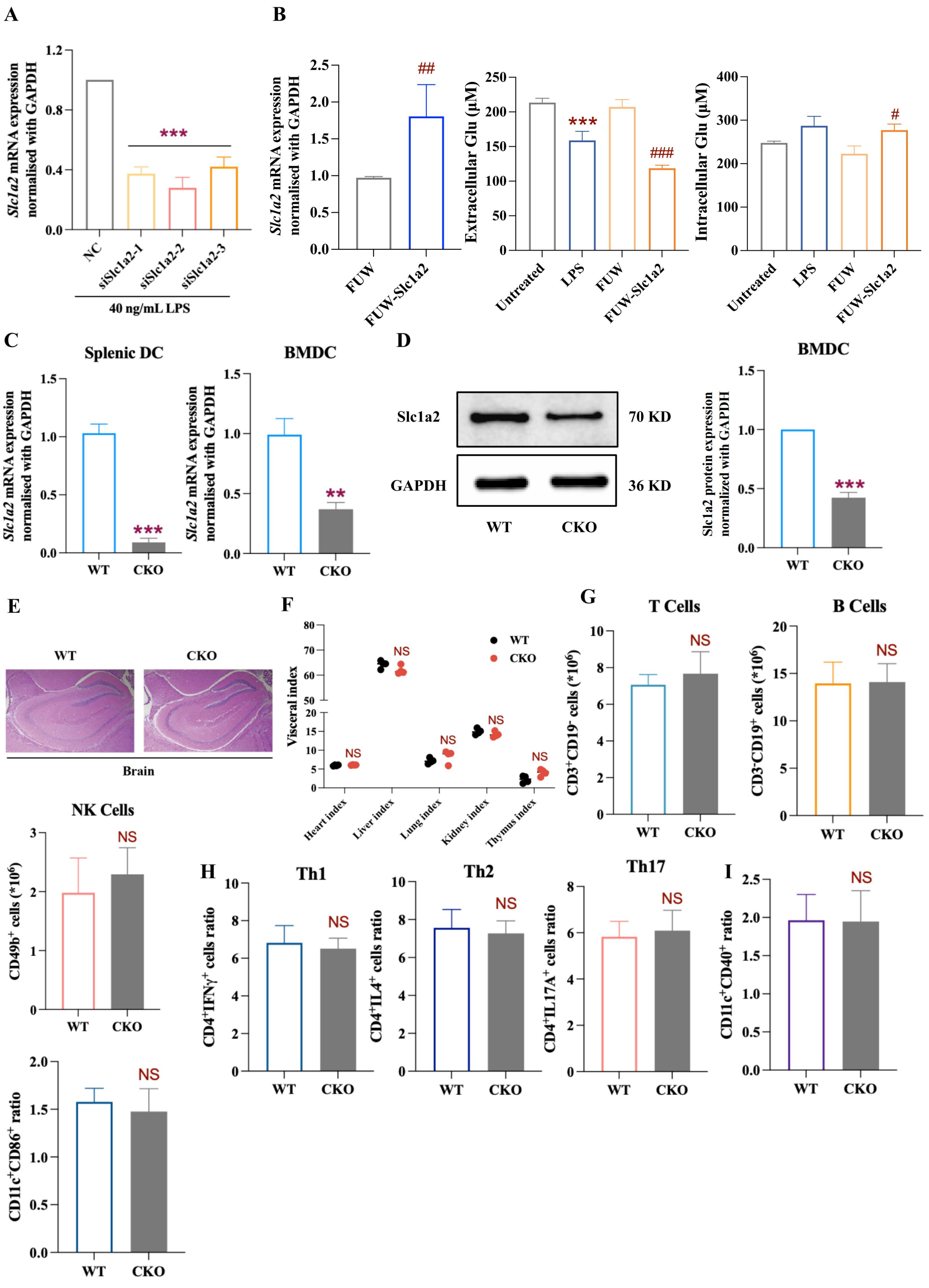

Supplementary Figure S1. A qRT-PCR analysis of Slc1a2 expression in BMDCs transfected with three different set of Slc1a2 silencers (si-Slc1a2-1, 2, 3) or a control silencer (NC), n=4/group, *: all groups compared to the NC group. **B** Intracellular and extracellular Glu content assay after transfection of Slc1a2 overexpression plasmid (FUW-Slc1a2) or a control vector (FUW). n=3/group, *: LPS groups compared to the Untreated group. #: FUW-Slc1a2 group compared to the FUW group. **C** qRT-PCR analysis of Slc1a2 expression in splenic DCs and BMDCs from $Slc1a2^{fl/fl}Itgax\text{-}cre^{+}$ (CKO) and $Slc1a2^{fl/fl}Itgax\text{-}cre^{-}$ (WT) mice. n=4/group. **D** Western blot analysis of Slc1a2 expression in WT/CKO-derived BMDCs. n=3/group. **E** Representative images of brain tissue sections stained with hemoglobin and eosin. **F** Individual organ indices in CKO and WT mice. n=3/group, NS, not significant. **G-I** Percentage of indicated immune cells (T, B, NK cells) (**G**), (Th1, Th2, Th17 cells) (**H**) or mDCs (**I**) in splenocytes from CKO and WT mice, NS, not significant. Data are presented as mean ± SD. Statistical analysis was performed using two-tailed Student's t-test (**B**, **C**, **D**, **F**, **G**, **H**, **I**), One-Way ANOVA (**A**). $^{\#}P < 0.05$, $^{**,\#\#}P < 0.01$, $^{***,\#\#\#}P < 0.001$.

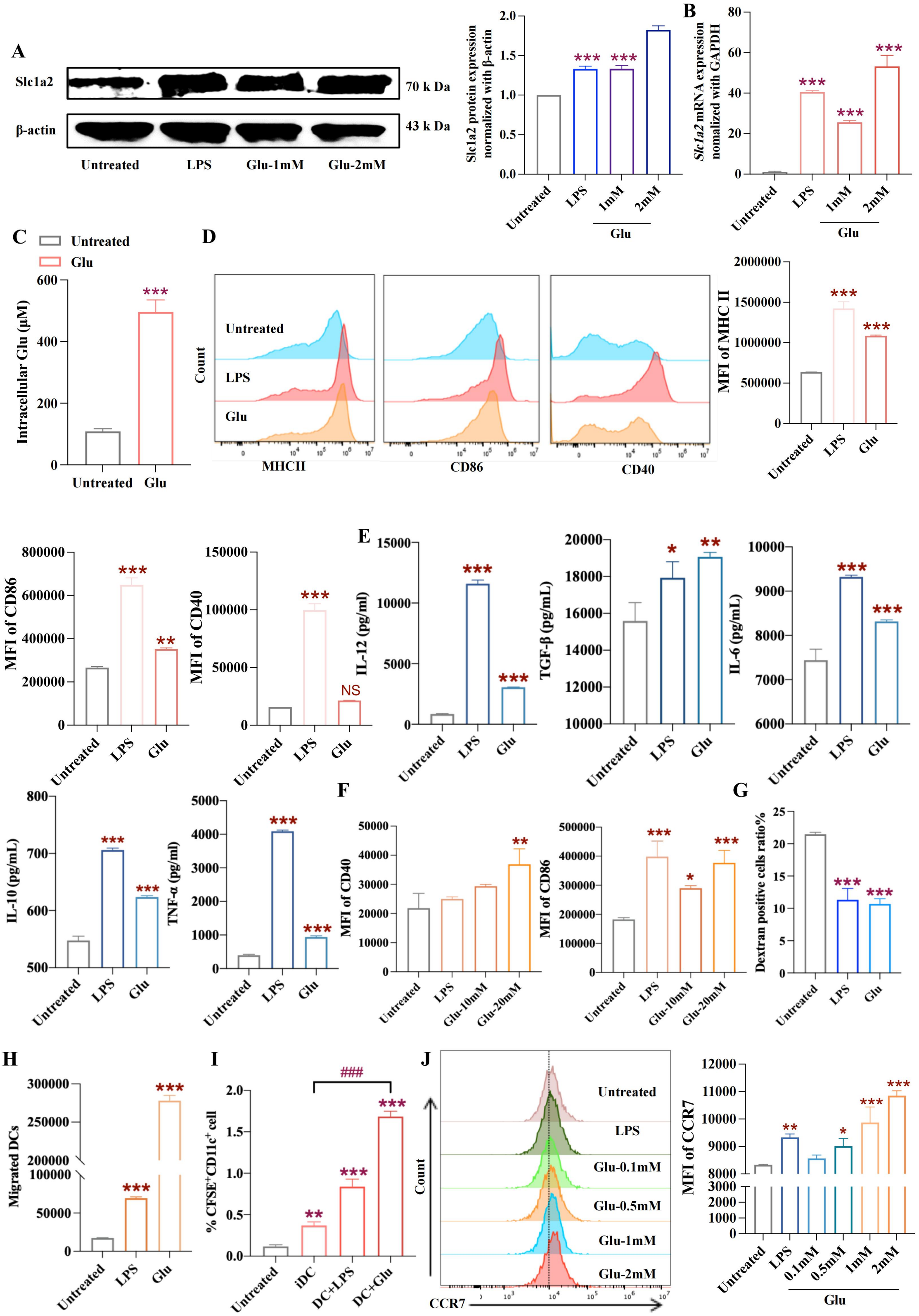
A
Slc1a2
β-actin
70 k Da
43 k Da
Untreated
LPS
Glu-1mM
Glu-2mM
Slc1a2 protein expression normalized with β-actin
B
Slc1a2 mRNA expression nomalized with GAPDH
Glu
C
Untreated
Glu
Intracellular Glu (μM)
D
Count
MHCII
CD86
CD40
MFI of MHC II
MFI of CD86
MFI of CD40
NS
E
IL-12 (pg/ml)
TGF-β (pg/mL)
IL-6 (pg/mL)
IL-10 (pg/mL)
TNF-α (pg/ml)
F
Glu-10mM
Glu-20mM
G
Dextran positive cells ratio%
H
Migrated DCs
I
% CFSE+CD11c+ cell
iDC
DC+LPS
DC+Glu
###
J
CCR7
Glu-0.1mM
Glu-0.5mM
Glu-1mM
Glu-2mM
MFI of CCR7

Supplementary Figure S2. **A** Protein levels of Slc1a2 in BMDCs, treatment with LPS, 1 mM Glu, and 2 mM Glu, n=3/group. **B** Levels of Slc1a2 mRNA in BMDCs, treatment with LPS, 1 mM Glu, and 2 mM Glu, n=4/group. **C** Intracellular Glu levels in BMDCs, treated with 2 mM Glu, n=3/group. **D** Expression of surface molecule MHCII, CD86, and CD40 in BMDCs treated with LPS and Glu, n=3/group. **E** Levels of proinflammatory cytokines (IL-12, TGF-β, IL-6, IL-10, TNF-α) in the culture supernatant of LPS/Glu-activted BMDCs, n=3/group. **F** Expression of surface molecules CD40 and CD86 in the $Cd11c^+$ $MHCII^+$ subset of popliteal lymph nodes, n=3/group. **G** Proportion of Dextran-positive cells after LPS/Glu-activated BMDCs were co-incubated with FITC-Dextran for 2 h, n=3/group. **H** LPS/Glu-triggered migration of BMDCs, analyzed in a Transwell chamber, n=3/group. **I** LPS/Glu-triggered migration of BMDCs, analyzed by popliteal lymph nodes, n=3/group, *: all groups compared to the untreated group, #: iDCs (immature DCs) group compared to the DC+Glu group. **J** Expression of chemokine receptor CCR7 in BMDCs after treatment with different concentrations of Glu, n=3/group. Data are presented as mean ± SD. Statistical analysis was performed using two-tailed Student's t-test (**C**), One-Way ANOVA (**A**, **B**, **D**, **E**, **F**, **G**, **H**, **J**), two-tailed Student's t-test and One-Way ANOVA (**I**). $^{*}P < 0.05$, $^{**}P < 0.01$, $^{***}P < 0.001$.

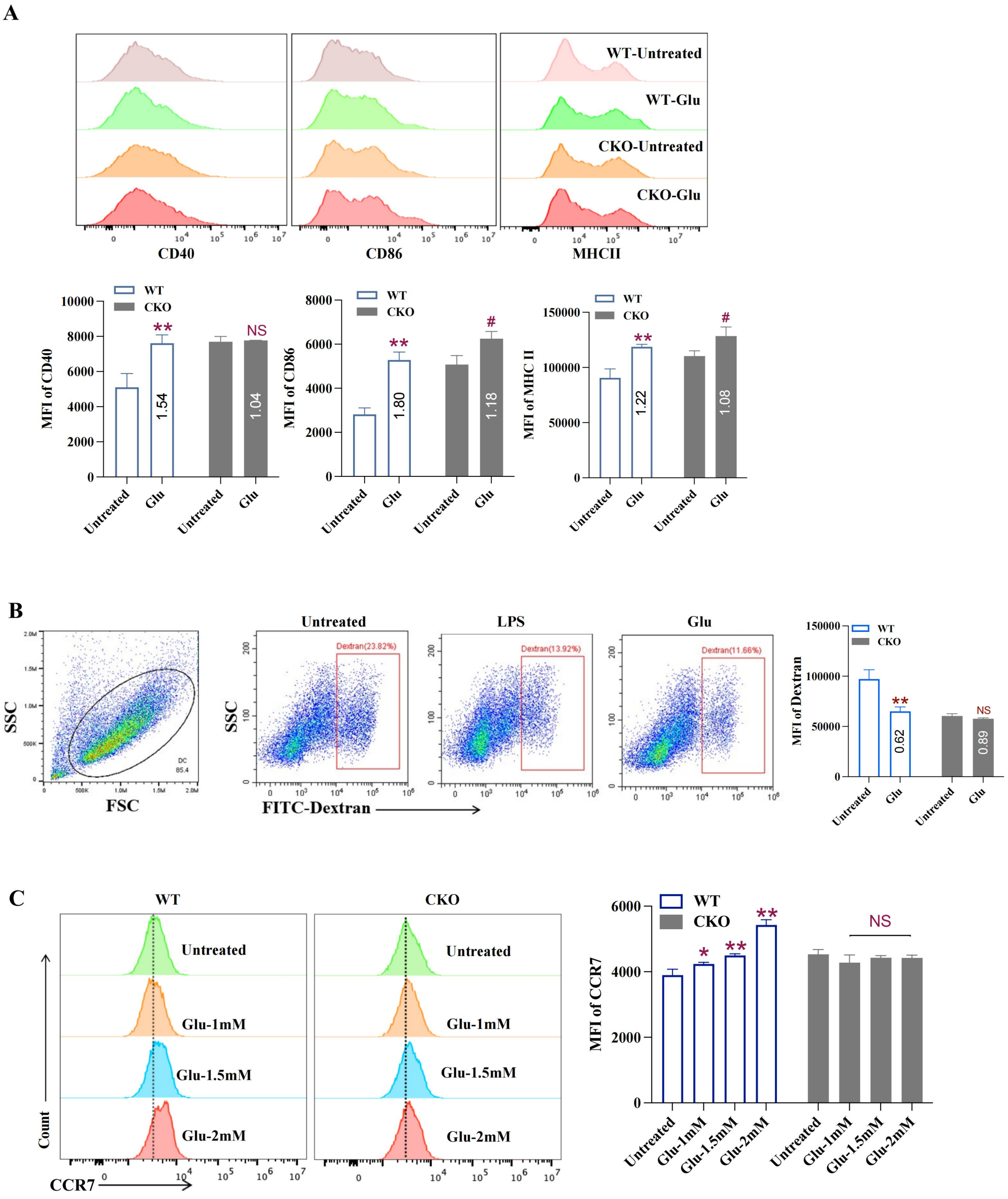

Supplementary Figure S3. A Expression of surface molecules CD40, CD86, and MHCII on WT/CKO-derived BMDCs treatment with Glu, n=3/group, *: WT-derived DCs, Glu group compared to the untreated group, #: CKO-derived DCs, Glu group compared to the untreated group. **B** Proportion of Dextran-positive cells and MFI of FITC-Dextran in Glu-activated WT/CKO-derived BMDCs, n=3/group, *: WT-derived BMDCs, Glu group compared to the untreated group, NS, not significant. **C** Expression of chemokine receptor CCR7 in WT/CKO-derived BMDCs treatment with Glu, n=3/group, *: WT-derived DCs, Glu group compared to the untreated group, NS, not significant. Data are presented as mean ± SD. Statistical analysis was performed using two-tailed Student's t-test (**A**, **B**), One-Way ANOVA (**C**). $^{*,\#}$P < 0.05, **P < 0.01.

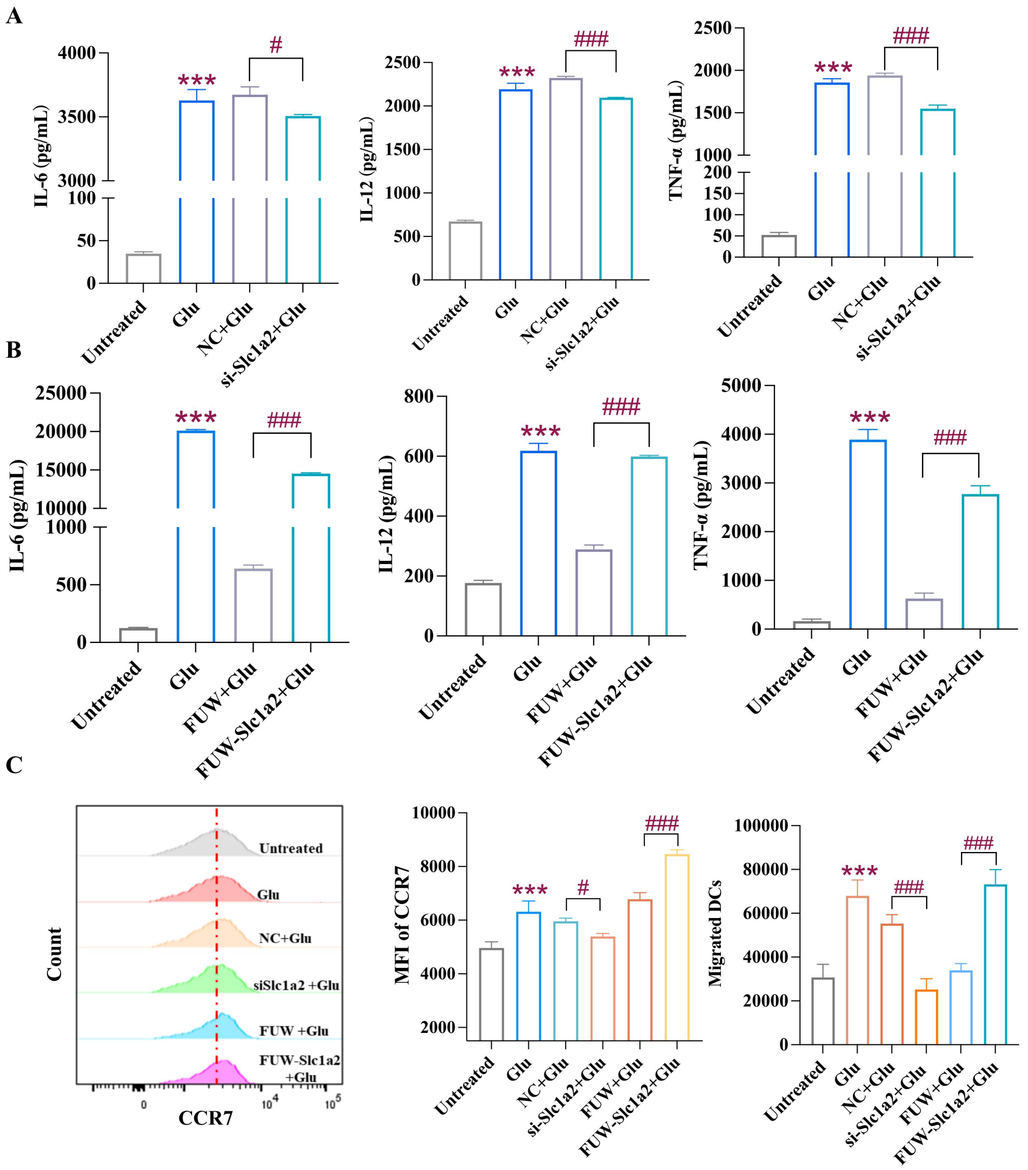

Supplementary Figure S4. A-B Levels of IL-6, IL-12, TNF-α in DC culture supernatants after transfection of Slc1a2 silencers (**A**) or overexpression plasmids (**B**), n=3/group, *: Glu groups compared to the Untreated group, #: si-Slc1a2/FUW-Slc1a2+Glu group compared to the NC/FUW+Glu group. **C** Expression of CCR7 and migrated cells in Transwell chembers after transfection of Slc1a2 silencers or overexpression plasmids, n=3/group, *: Glu groups compared to the Untreated group. #: si-Slc1a2/FUW-Slc1a2+Glu group compared to the NC/FUW+Glu group. Data are presented as mean ± SD. Statistical analysis was performed using two-tailed Student's t-test. $^{\#}P < 0.05$, $^{***,\#\#\#}P < 0.001$.

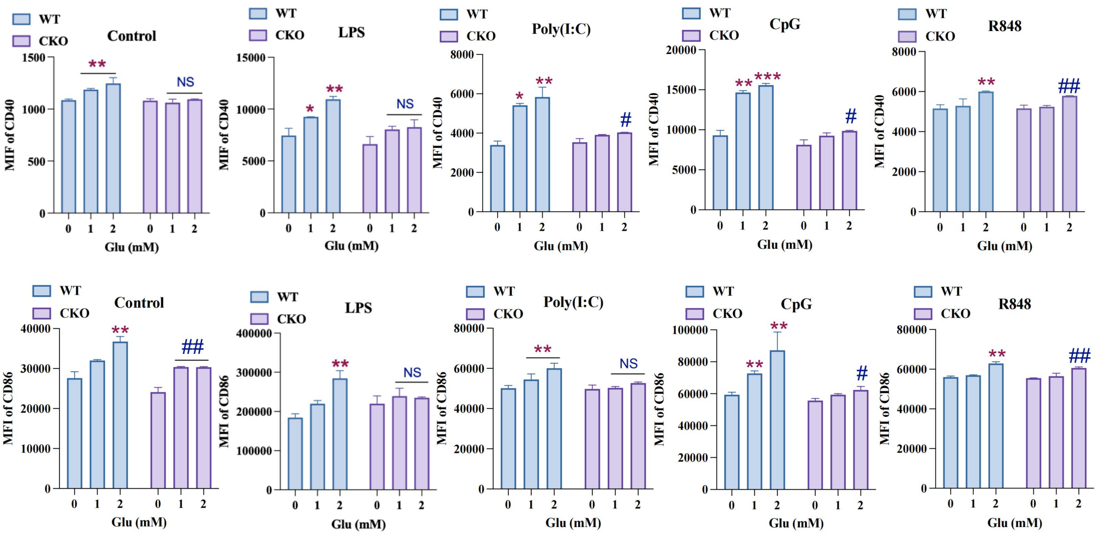

Supplementary Figure S5. Expression of surface molecules CD40 and CD86 on WT/CKO-derived BMDCs treatment with 0, 1, 2 mM Glu + TLR agonists, n=3/group, *: WT-derived DCs, all groups compared to the untreated group, #: CKO-derived DCs, all groups compared to the untreated group, NS, not significant. Data are presented as mean ± SD. Statistical analysis was performed using One-Way ANOVA. $^{*,\#}P < 0.05$, $^{**,\#\#}P < 0.01$, $^{***}P < 0.001$.

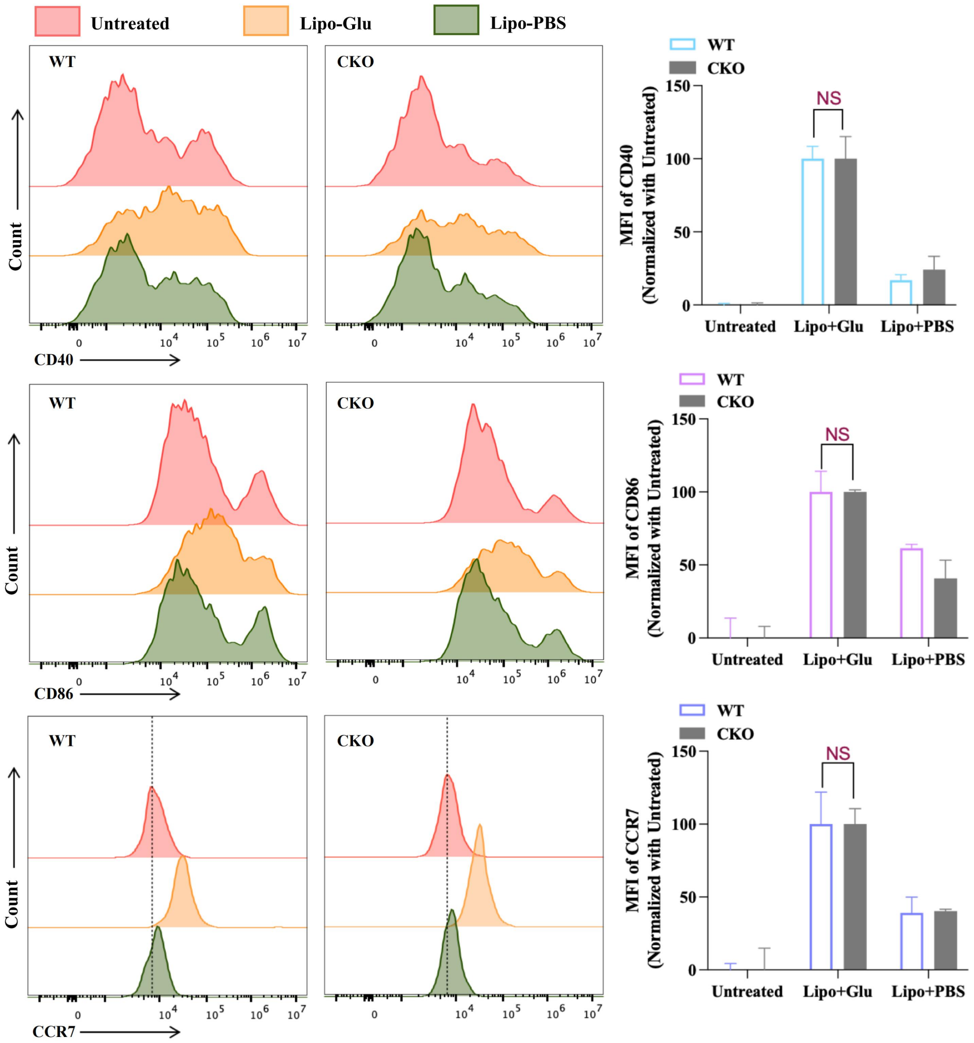

Supplementary Figure S6. Liposome-encapsulated Glu (Lipo-Glu) and Liposome-encapsulated PBS (Lipo-PBS), treated WT/CKO-derived BMDCs, the expression of maturation- and migration-related surface molecules CD40, CD86, and CCR7 was assessed by flow cytometry. n=3/group. NS, not significant.

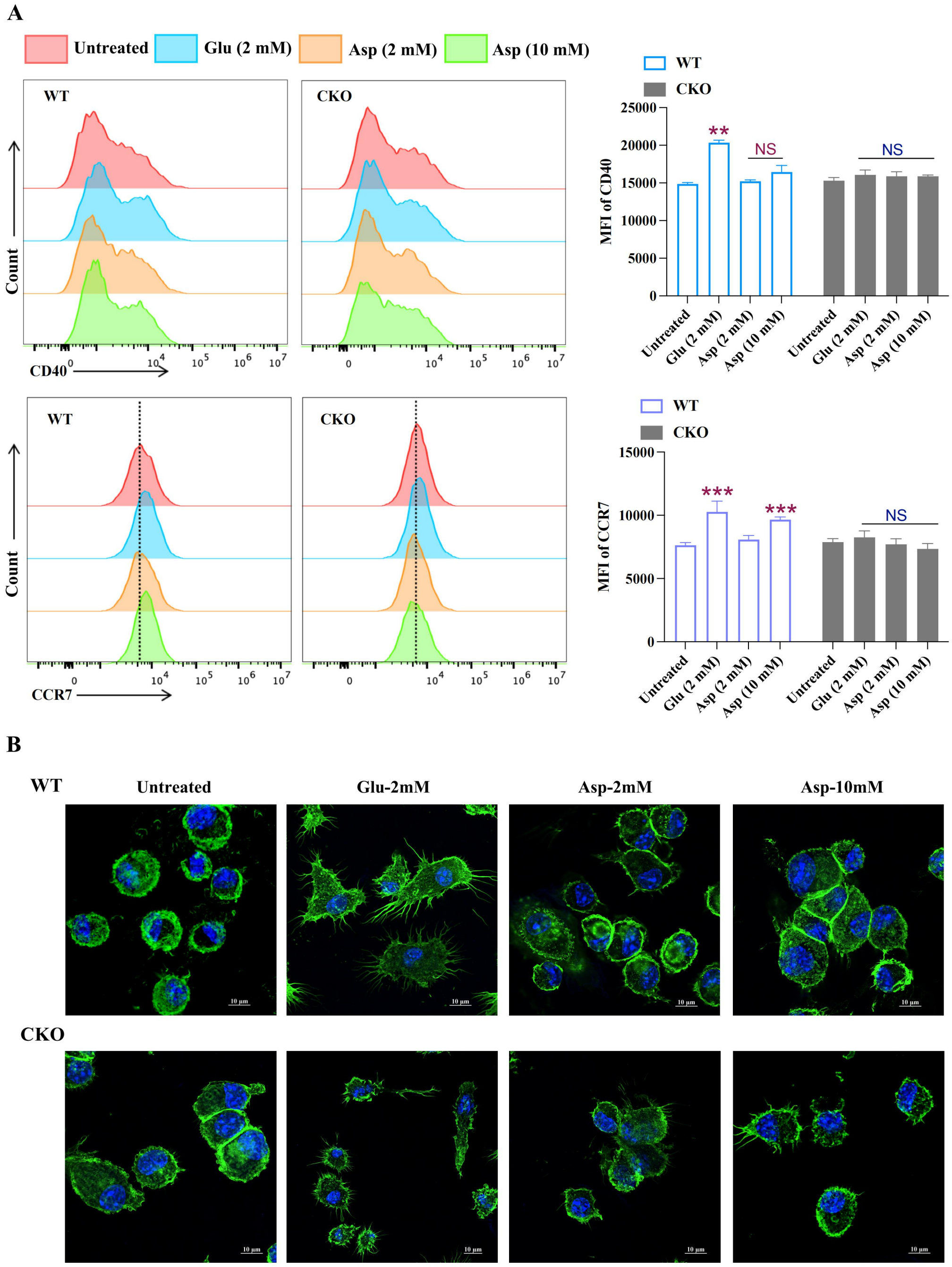

Supplementary Figure S7. A Expression of surface molecules CD40 and CCR7 on WT/CKO-derived BMDCs treatment with Glu (2 mM), Asp (2 mM), Asp (10 mM), n=3/group, *: WT-derived DCs, all groups compared to the untreated group, #: CKO-derived DCs, all groups compared to the untreated group, NS, not significant. **B** After treatment with Glu (2mM), Asp (2 mM), and Asp (10 mM), immunofluorescence staining revealed the cytoskeletal morphology of WT/CKO-derived BMDCs. DNA (blue) was stained with DAPI, F-actin (green). Scale bars, 10 μm. Data are presented as mean ± SD. Statistical analysis was performed using One-Way ANOVA. $^{**}P < 0.01$, $^{***}P < 0.001$.

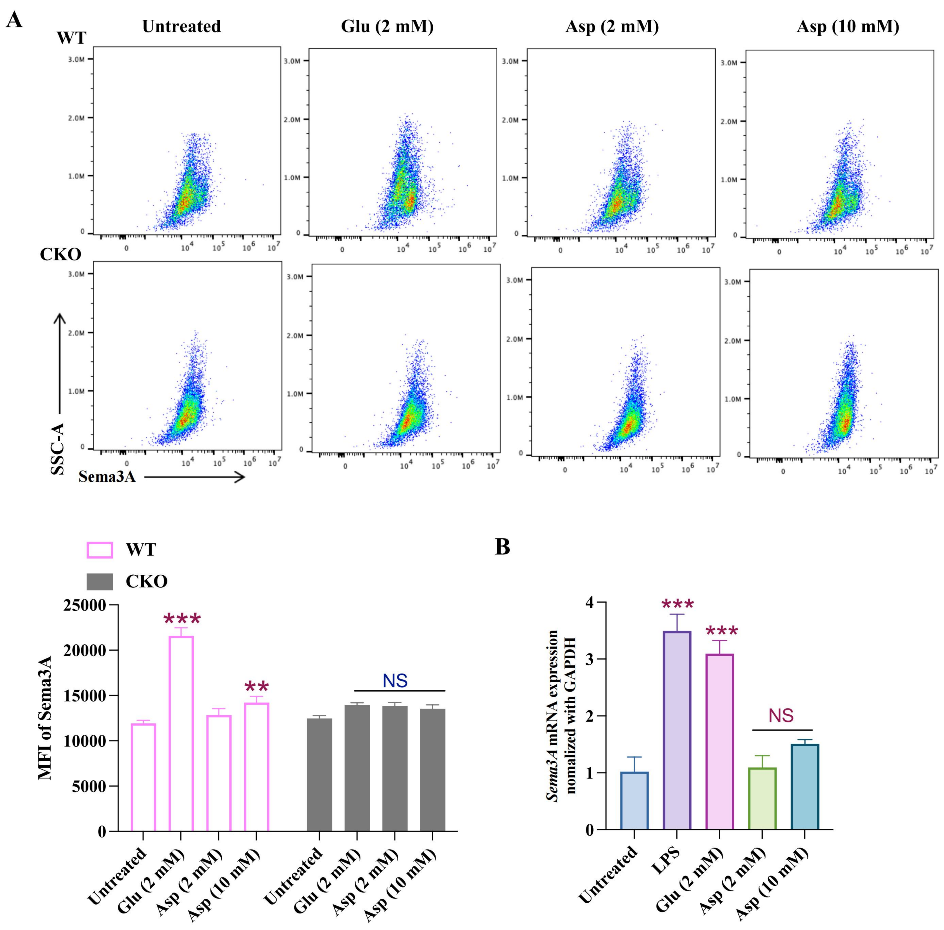

Supplementary Figure S8. A Expression of Sema3A on WT/CKO-derived BMDCs treatment with Glu (2 mM), Asp (2 mM), Asp (10 mM), n=3/group, *: WT-derived DCs, all groups compared to the untreated group, #: CKO-derived DCs, all groups compared to the untreated group, NS, not significant. **B** qRT-PCR analysis of Sema3A mRNA expression after LPS, Glu (2 mM), Asp (2 mM), Asp (10 mM) treatment in wild-type BMDCs, n=4/group, NS, not significant. Data are presented as mean ± SD. Statistical analysis was performed using One-Way ANOVA. $^{**}P < 0.01$, $^{***}P < 0.001$.

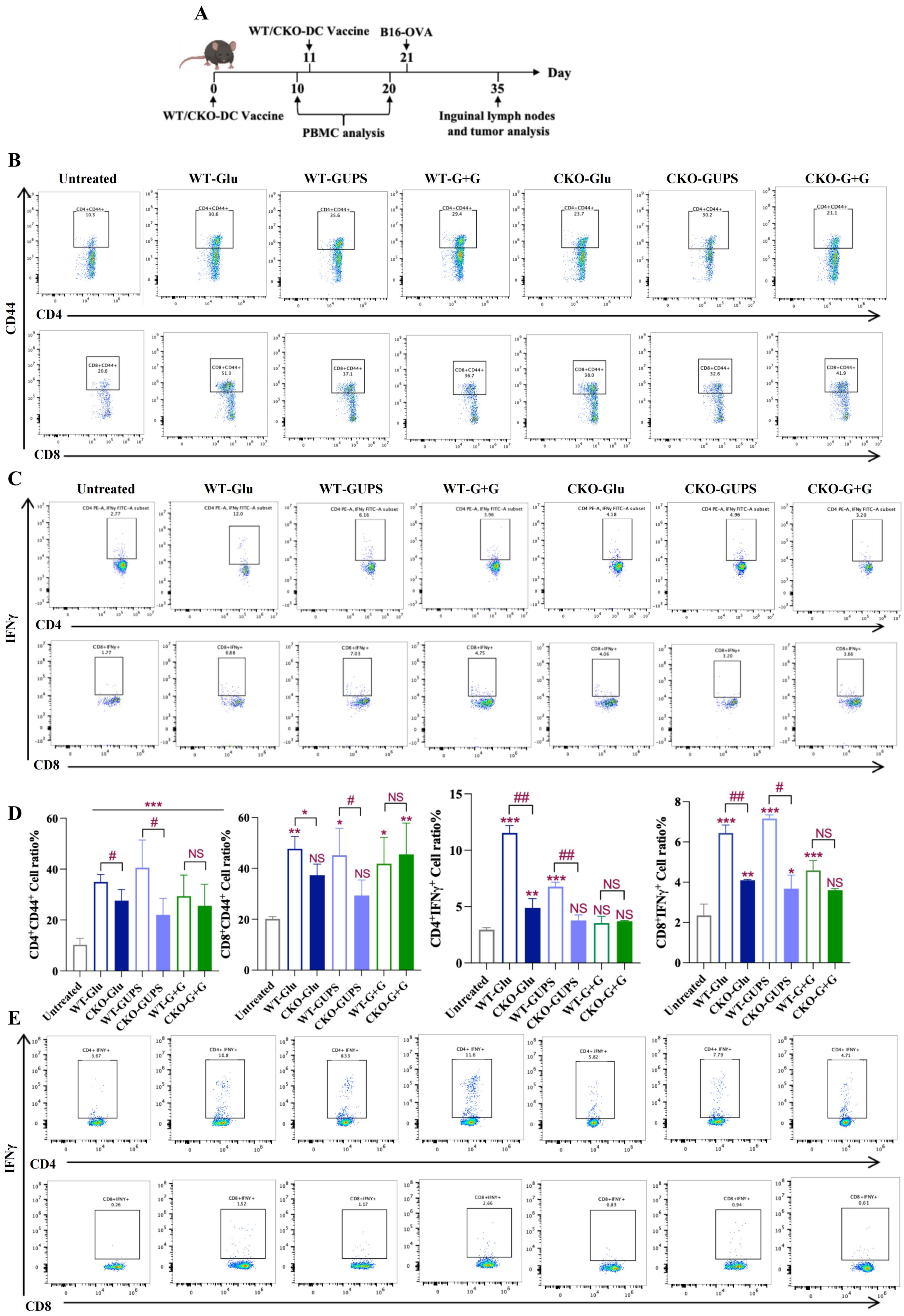
A
WT/CKO-DC Vaccine
11
B16-OVA
21
Day
0
10
20
35
WT/CKO-DC Vaccine
PBMC analysis
Inguinal lymph nodes and tumor analysis
B
Untreated
WT-Glu
WT-GUPS
WT-G+G
CKO-Glu
CKO-GUPS
CKO-G+G
CD44
CD4
CD8
C
IFNγ
D
CD4+CD44+ Cell ratio%
CD8+CD44+ Cell ratio%
CD4+IFNγ+ Cell ratio%
CD8+IFNγ+ Cell ratio%
E

Supplementary Figure S9. A Timeframe for WT/CKO-derived BMDC preventive vaccines effect in the B16-OVA tumor model, n=3/group. **B-D** Proportion of activated $CD4^+$/$CD8^+$ T cells and CTL/Th1 cells in PBMC after the first treatment with WT/CKO-derived BMDC vaccines. **E** Proportion of CTL and Th1 cells in PBMC after the second treatment with WT/CKO-derived BMDC vaccines, n=3/group, *: all groups compared to the untreated group, #: Under the same treatment, WT and CKO-derived BMDC vaccines were compared, NS, not significant. Data are presented as mean ± SD. Statistical analysis was performed using two-tailed Student's t-test and One-Way ANOVA. $^{*,\#}P < 0.05$, $^{**,\#\#}P < 0.01$, $^{***}P < 0.001$.
